# ﻿Japanese species of *Ormosia* Rondani (Diptera, Limoniidae): revision of the subgenera *Oreophila* Lackschewitz and *Parormosia* Alexander

**DOI:** 10.3897/zookeys.1132.86022

**Published:** 2022-11-29

**Authors:** Daichi Kato, Kozo Watanabe, Levente-Péter Kolcsár

**Affiliations:** 1 Echigo-Matsunoyama Museum of Natural Science, ‘Kyororo’, 1712-2 Matsunoyama-Matsuguchi, Tôkamachi, 942-1411, Japan Echigo-Matsunoyama Museum of Natural Science Tôkamachi Japan; 2 Center for Marine Environmental Studies (CMES), Ehime University, Matsuyama, Ehime 790-8577, Japan Ehime University Matsuyama Japan

**Keywords:** Crane flies, new species, subapterous, taxonomy, terminalia, Tipuloidea

## Abstract

Japanese species of the subgenera *Oreophila* Lackschewitz and *Parormosia* Alexander of the genus *Ormosia* Rondani (Limoniidae) are revised. Two new species Ormosia (Oreophila) komazawai Kato & Kolcsár, **sp. nov.** and Ormosia (Parormosia) phalara Kato & Kolcsár, **sp. nov.** are described. The identities of all Japanese species of the two subgenera are clarified and redescribed with images of habitus and wings, and drawings of male and female terminalia. The first DNA barcode sequences of the species Ormosia (Parormosia) diversipes Alexander and Ormosia (Parormosia) phalara Kato & Kolcsár, **sp. nov.** are also provided. A key to, and distribution maps of, the Japanese species are provided.

## ﻿Introduction

*Ormosia* Rondani (1856) is a relatively large genus of the subfamily Chioneinae (family Limoniidae) and includes 224 species worldwide (Oosterbroek 2022). Twelve fossil species of the genus have been described from the Baltic amber (Podėnas 1999). The adults are characterized by wing cells covered with hair-like setae and well-developed meron separating mid and hind coxae. Similar morphological characters occur in genera *Amphineurus* Skuse, *Rhypholophus* Kolenati, *Scleroprocta* Edwards, Molophilus (Trichomolophilus) Alexander, and *Trichotrimicra* Alexander, but *Ormosia* is distinguished from these by the following combination of characters: wing vein M_3_ joining vein M_1+2_ or vein M_4_ beyond cord; male terminalia rotated by 90–180 degrees; clasper of gonostylus not dilated or bifid on distal part; aedeagus simple, not bifid at tip. The genus is divided into four subgenera: *Neserioptera* Alexander (2 spp., Afrotropical); *Oreophila* Lackschewitz (17 spp., Nearctic, Palaearctic and Oriental); *Ormosia* (182 spp., Nearctic, Palaearctic and Oriental); *Parormosia* Alexander (23 spp., Eastern Palaearctic, Nearctic and Oriental).

Immature stages of *Ormosia* spp. are known to inhabit wet soil or moist dead woods (Alexander 1920; Brinkmann 1991; Krivosheina and Krivosheina 2011), but our knowledge on the biology of immature stages is still sporadic.

Twenty-two species of *Ormosia* are reported from Japan, represented by two species of each of the subgenera *Oreophila* and *Parormosia* and by 18 species belong to the subgenus Ormosia s. s. (Nakamura 2014; Oosterbroek 2022).

As a first step in clarifying the taxonomy of the genus *Ormosia* in Japan, species of the subgenera *Oreophila* and *Parormosia* are revised in this paper. In addition to redescribing the four already known species, viz. Ormosia (Oreophila) confluenta Alexander, 1922, Ormosia (Oreophila) sootryeni (Lackschewitz, 1935), Ormosia (Parormosia) diversipes Alexander, 1919, and Ormosia (Parormosia) nippoalpina Alexander, 1941, two new species are described. Images of the habitus and wings, drawings of the male terminalia, and a key to and distribution maps of the Japanese species are provided. DNA barcode sequences of the species Ormosia (Parormosia) diversipes Alexander and Ormosia (Parormosia) phalara Kato & Kolcsár, sp. nov. are uploaded to The Barcode of Life Data System (BOLD) (Ratnasingham and Hebert 2007).

## ﻿Materials and methods

Specimens were collected using insect nets, Malaise traps or at light traps and preserved in 70% or 90% ethanol or pinned. Overall descriptions of the species were based on the observations through a Leica S APO and Zeiss Stemi 508 stereomicroscopes. Male terminalia were put in vials filled with a solution of 10% KOH and the vials were heated in hot water for several minutes. Then the terminalia were rinsed in a solution of 70% ethanol with 3% acetic acid for neutralization, transferred to glycerol for examination and drawing, and preserved in genitalia tubes filled with glycerol. The genitalia tubes were pinned below the body remains. Drawings were made using the stereomicroscope equipped with a grid eyepiece micrometer. Terminology followed Cumming and Wood (2017) for general description, de Jong (2017) for wing venation, Ribeiro (2008) for male terminalia, and Starý and Brodo (2009) for female terminalia. General distributions of species were referred to Catalogue of the Craneflies of the World (Oosterbroek 2022). For the rotated male terminalia in *Ormosia*, directions as “dorsal” and “ventral” are used correspondingly to the tergal and sternal positions.

Specimens from the following depositories were examined:

**BLKU**Biosystematics Laboratory, Kyushu University, Japan;

**CKLP** Private collection of Levente-Péter Kolcsár;

**CMK** Komazawa’s Private Collection, Asahikawa City, Hokkaido, Japan;

**EUMJ**Ehime University Museum, Matsuyama, Japan;

**USNM**National Museum of Natural History, Smithsonian Institution, Washington, D.C., USA.

Mitochondrial DNA was extracted using DNeasy Blood & Tissue kits (Qiagen GmbH, Hilden, Germany). The 658 bp fragment of COI gene was amplified using LCO-1490 and HCO-2198 primers (Folmer et al. 1994). The PCR products were sequenced by Eurofins Operon (Tokyo, Japan). Forward and reverse reads were assembled using CodonCode Aligner v 3.5 (Codon Code Corporation, Dedham, USA). Consensus barcode sequences were submitted to BoldSystems (http://www.boldsystems.org).

### ﻿Abbreviations

**ea** ejaculatory apodeme;

**ad** aedeagus;

**as** aedeagal sheath;

**cg** clasper of gonostylus;

**cr** cercus;

**dcg** dorsal arm of clasper of gonostylus;

**hv** hypogynial valve;

**ib** interbase;

**ga** gonocoxal apodeme;

**gc** gonocoxite;

**gf** genital fork;

**go** genital opening;

**lag** lateral arm of genital fork;

**lg** lobe of gonostylus;

**ml** mesal-apical lobe of interbase;

**pm** paramere;

**sd** spermathecal duct;

**sp** sperm pump;

**s** sternite;

**t** tergite;

**vcg** ventral arm of clasper of gonostylus.

## ﻿Results

### ﻿Taxonomic treatment

#### ﻿Genus Ormosia

Taxon classificationAnimaliaDipteraLimoniidae

Rondani, 1856

3CBEF6F7-921C-5D3E-96A7-8F552D8DA04C

##### Type species.

*Eriopteranodulosa* Macquart, 1826 (Macquart 1826) by original designation.

#### ﻿ Subgenus Oreophila

Taxon classificationAnimaliaDipteraLimoniidae

Lackschewitz, 1935

03C7D51B-B625-5A23-AA3C-9988B8A3B919

##### Type species.

*Rypholophusbergrothi* Strobl, 1895 (Strobl 1895) by original designation.

##### Note.

This subgenus includes 17 species worldwide, prior to this article (7 Palaearctic, 7 Nearctic, and 3 Oriental species) (Oosterbroek 2022).

#### ﻿ Ormosia (Oreophila) confluenta

Taxon classificationAnimaliaDipteraLimoniidae

Alexander, 1922

12777ED8-864B-5DC6-BD1A-68DC255C9E78

**Figure 1.**
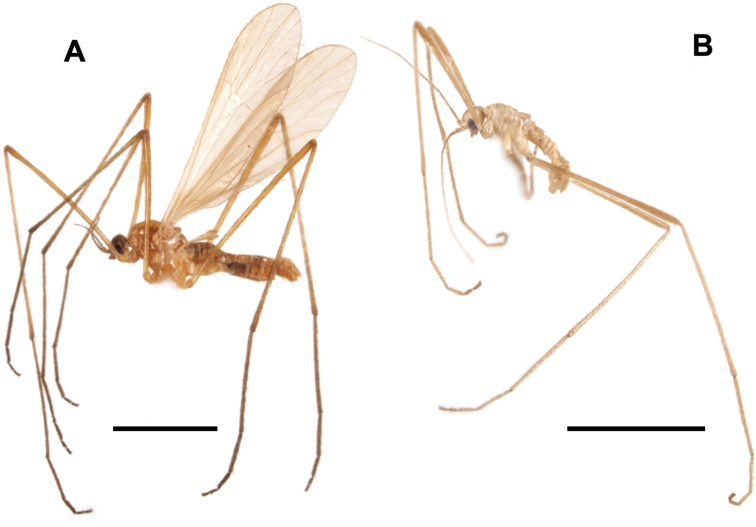
Habitus of male **A**Ormosia (Oreophila) confluenta Alexander, 1922 **B**Ormosia (Oreophila) komazawai Kato & Kolcsár, sp. nov. Scale bars: 2 mm.

**Figure 2.**
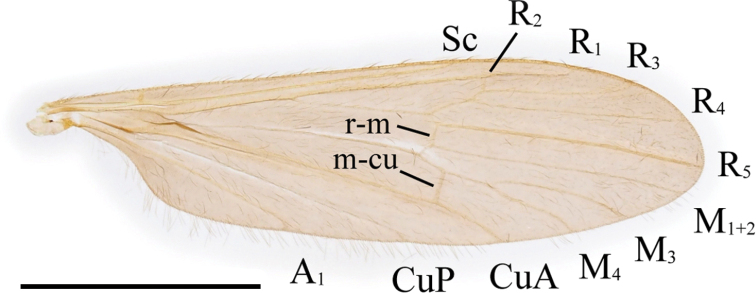
Wing of Ormosia (Oreophila) confluenta Alexander, 1922. Scale bar: 2 mm.

**Figure 3.**
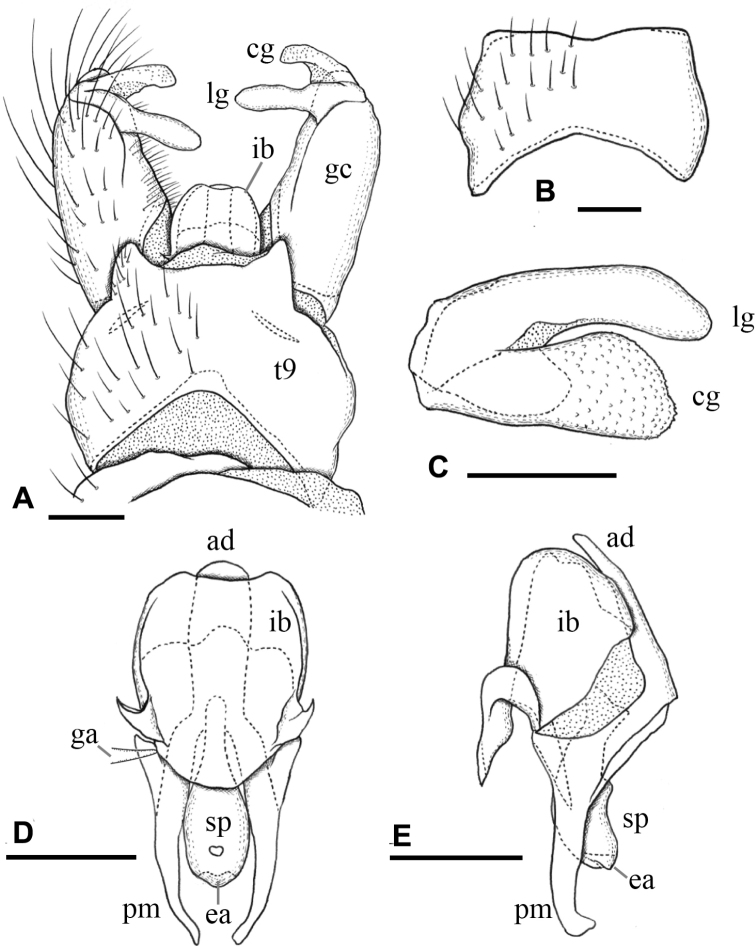
Male terminalia of Ormosia (Oreophila) confluenta Alexander, 1922 **A** dorsal view **B** sternite 9, ventral view **C** gonostylus, outer surface **D** aedeagal complex, dorsal view (left gonocoxal apodeme omitted) **E** aedeagal complex, lateral view (left = dorsal). Scale bars: 0.1 mm.

**Figure 4.**
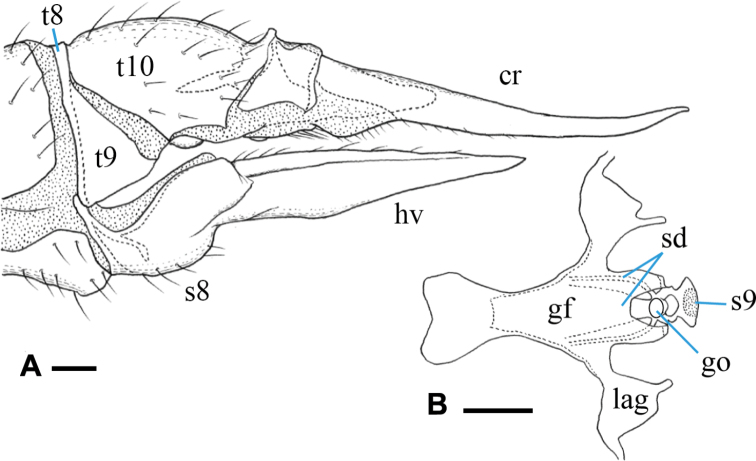
Female terminalia of Ormosia (Oreophila) confluenta Alexander, 1922 **A** lateral view **B** genital frame, ventral view (left = anterior). Scale bars: 0.1 mm.

**Figure 5.**
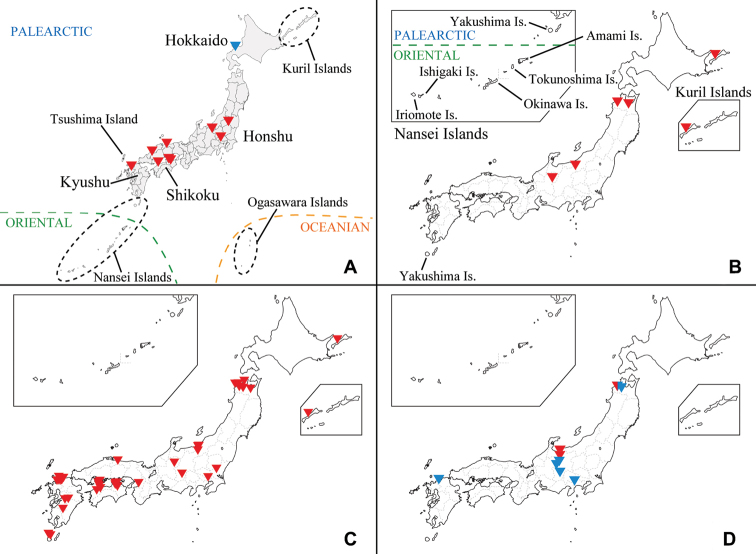
Distribution map of Japanese species of Ormosia (Oreophila) and Ormosia (Parormosia). **A**Ormosia (Oreophila) confluenta Alexander, 1922 (red), Ormosia (Oreophila) komazawai Kato & Kolcsár, sp. nov. (blue) **B**Ormosia (Oreophila) sootryeni Lackschewitz, 1935 **C**Ormosia (Parormosia) diversipes Alexander, 1919 **D**Ormosia (Parormosia) nippoalpina Alexander, 1941 (red), Ormosia (Parormosia) phalara Kato & Kolcsár, sp. nov. (blue).

Figs 1A , 2 , 3 , 4 , 5A

Ormosia confluenta in Alexander 1922: 183: original description (type locality: Japan, Honshu, Ôsaka, Mt. Minomo).
Ormosia (Ormosia) confluenta in Alexander 1953a: 71: faunistic record.
Ormosia (Oreophila) confluent in Nakamura 2002: 169: faunistic record; Nakamura 2014: 31: distribution; Oosterbroek 2022: distribution.

##### Type material examined.

***Paratype*.** Japan • ♀; Honshu, Mt. Minomo; 4 May 1921; K. Takeuchi leg.; USNM.

##### Non-type material examined.

Japan • 2 ♂; Honshu, Nagano, Ueda-shi, Sanada-machi-Osa, Kakuma Valley; 36.45378°N, 138.36592°E; alt. 1050 m; 16 May 2012; D. Kato leg.; BLKU. • 2 ♂; Honshu, Tokyo, near Tokyo, Mt. Mitake; 10 May 1931; B. Oda leg.; USNM. • 1 ♂; Honshu, Okayama, Maniwa-shi, Hiruzen-Shimotokuyama; 35.32931°N, 133.59725°E; alt. 780 m; 1 May 2016; D. Kato leg.; BLKU. • 1 ♀; Honshu, Hiroshima, Akiôta-chô, Yokogô; 34.59419°N, 132.14497°E; alt. 890 m; 18 May 2015; D. Kato leg.; BLKU. • 1 ♂; Shikoku, Tokushima, Mt. Tsurugi; alt. 1400–1950 m; 31 May 1950; Issiki and Ito leg.; USNM. • 1 ♂; Shikoku, Tokushima, Miyoshi-shi, Higashiiya-Ochiai, near Matsuogawa Dam; 33.96478°N, 133.93908°E; alt. 900 m; 15 May 2015; • 1 ♂, 1 ♀; same locality; 30 Apr. 2016; D. Kato leg., BLKU. • 6 ♂, 1 ♀; Shikoku, Tokushima, Miyoshi-shi, Higashiiya-Sugeoi, near Nagoro Dam; 33.85182°N, 134.0234°E; alt. 920 m; 29 Apr. 2016; • 3 ♂; same locality; 30 Apr. 2016; D. Kato leg. BLKU. • 4 ♂, 1 ♀; Shikoku, Ehime, Kumakogen, River Myogadani, springs; 33.56701°N, 132.9344 °E; 1420 m; 8 May 2022; L.-P. Kolcsár leg.; CKLP. • 1 ♂; Kyushu, Saga, Karatsu-shi, Kyuragi-Hirano, Mt. Sakurei-zan; 33.35701°N, 130.07038°E; alt. 860 m; 26 Apr. 2015; D. Kato leg.; BLKU.

##### Diagnosis.

General coloration yellow to pale brown (Fig. 1A). Vertex often pale brownish grey. Antenna dark brown except scape. Wing yellowish tinged, unpatterned. Legs distal to mid-tibiae gradually becoming dark brown towards tips. Male terminalia: tergite 9 bearing pair of triangular lobes at caudal margin. Gonocoxite slightly produced beyond base of clasper of gonostylus. Clasper of gonostylus wider apically, 3/4 length of lobe of gonostylus, distal part 2× as wide as that of lobe of gonostylus. Interbases fused medially into roundish sac-like plate, without mesal-apical lobe. Female terminalia with cercus almost straight, slightly upcurved on distal part. Genital frame with lateral arm of genital fork roughly triangular, situated at posterior 1/3 of genital fork, with finger-shaped lobe on posterior end. Sternite 9 very small, fan-shaped.

##### Redescription.

**Male**. Body length 2.9–4.2 mm, wing length 4.1–5.9 mm.

***Head***: covered with yellow to brown setae. Vertex dusky yellow to pale brownish grey, paler grey on anterior part, often widely dark brown on center of posterior part. Eyes small and widely separated, ~ 1/2 as wide as narrowest point of vertex, ~ 1/3 length of head including rostrum in dorsal view. Rostrum dusky yellow to pale brown, ~ 1/2 length of eye in lateral view. Palpus 5-segmented, 2/3 length of head, dusky yellow on basal two segments, dark brown on succeeding segments, palpomere 1 small, globular, palpomere 2 cylindrical, palpomeres 3–5 globular. Labellum dark brown. Antenna 15 to 16-segmented, relatively short, ~ 2× length of head; scape dusky yellow to pale brown, 2× as long as wide; pedicel dark brown, oval, 2/3 length of scape; flagellomeres dark brown, barrel-shaped, gradually decreasing in size toward apical segment; each flagellomere with ca. six verticils, longest one except in apical segment ~ 1.5× as long as each segment.

***Thorax***: covered with yellow setae. Antepronotum dusky yellow to pale brown; postpronotum pale yellow. Mesonotum subnitidous, pale brown to brown, lateral margin yellow, postero-outer corner of scutal lobe often yellow. Scutellum or mediotergite sometimes yellowish. Prescutal pit indistinctly present, oval to bacilliform. Tuberculate pit indistinctly present, situated slightly anterior to level of prescutal pit. Pleuron dusky yellow to yellow. Wing (Fig. 2) yellowish tinged, narrow, 3.5–4× as long as wide; stigma absent; Sc ending between level of forks of Rs and R_2+3+4_; crossvein sc-r indistinct or absent, situated on level of basal 1/3 of Rs if present; R_2+3+4_ 1/7–1/4 length of R_3_; R_2_ situated between fork of R_2+3+4_ and length of itself distal to it; M_4_ 1–1.5× as long as M_3+4_; wing margin between tips of CuP and A_1_ 1.3–2× as long as that between tips of CuP and CuA; A_1_ almost straight. Halter yellow, ~ 2/3 length of thorax. Legs yellow on coxae to femora; tibiae yellow basally, gradually turning to dark brown toward tips; tarsi dark brown (Fig. 1A).

***Abdomen***: yellow to yellowish ochreous, densely covered with yellow setae.

***Male terminalia*** (Fig. 3): Tergite 9 with pair of triangular lobes at caudal margin, ~ 1/3 length of middle of tergite 9; anterior margin of tergite 9 deeply and widely notched; tergite 9 slightly wider than long including caudal lobe (Fig. 3A). Sternite 9 almost straight at caudal margin (Fig. 3B). Gonocoxite almost same width in whole length, ca. as long as tergite 9, posteroventral margin slightly produced beyond base of clasper of gonostylus (Fig. 3A). Gonocoxal apodeme short, connected to anterolateral part of interbase (Fig. 3D). Clasper of gonostylus scabrous, darkened apically, 3/4 length of lobe of gonostylus, gradually wide distally, rounded at tip, distal part 2× as wide as that of lobe of gonostylus in apical view (Fig. 3C). Lobe of gonostylus long finger-shaped, ~ 1/2 length of gonocoxite, slightly curved, distal part flattened in apical view (Fig. 3C). Interbases fused medially into roundish sac-like plate, ca. as long as wide in dorsal view, posterior margin slightly concave, anterolateral part with short arm (Fig. 3D). Paramere roughly blade-shaped, ca. as long as interbase, anterior end curved inward (Fig. 3D). Aedeagus dorso-ventrally flattened, ~ 1/3 width of interbase, tip slightly beyond apex of interbase (Fig. 3D). Sperm pump long oval, anterior end reaching at anterior 1/3 of paramere (Fig. 3D, E). Ejaculatory apodeme poorly developed (Fig. 3D, E).

**Female**. Body length 5.0–6.2 mm, wing length 5.6–6.7 mm. Generally resembling male.

***Female terminalia*** (Fig. 4): yellow to yellowish ochreous, cercus and hypogynial valve amber-colored. Tergites 8 and 9 fused. Cercus almost straight, slightly upcurved on distal part, 1.5× as long as tergite 10; hypogynial valve ~ 1.7× as long as sternite 8, basal part distinctly wider than that of cercus, gradually narrowed on distal 1/2 toward tip, tip acute, ending near level of middle of cercus (Fig. 4A). Genital frame with genital fork, constricted at anterior 1/3, roughly heart-shaped on anterior end; lateral arm of genital fork roughly triangular distally, situated at anterior 2/3 of genital fork, with small finger-shaped lobe at posterior end; sternite 9 very small, narrower than posterior end of genital fork, fan-shaped distally, largely desclerotized on middle of posterior part. Three spermathecal ducts present, middle one very wide; spermathecae indistinct (Fig. 4B).

##### Distribution.

Japan (Honshu, Shikoku, and Kyushu) (Fig. 5A), Russia (Far East), and Kuril Is.

##### Remarks.

This species resembles a Nearctic species, Ormosia (Oreophila) flaveola (Coquillett, 1900) (Coquillett 1900), and an Eastern Palaearctic species known from North Korea and China, Ormosia (Oreophila) yankovskyi Alexander, 1940 (Alexander 1940a), but is differentiated from them by the following characters: femora entirely yellow (yellow basally with darker apical parts in Ormosia (Oreophila) flaveola and Ormosia (Oreophila) yankovskyi).

#### ﻿ Ormosia (Oreophila) komazawai

Taxon classificationAnimaliaDipteraLimoniidae

Kato & Kolcsár sp. nov.

20BE81C9-25C4-51AA-BD8B-69ADCF8F69AD

https://zoobank.org/60D987FD-CE30-4CC5-A590-638024C82CA9

**Figure 6.**
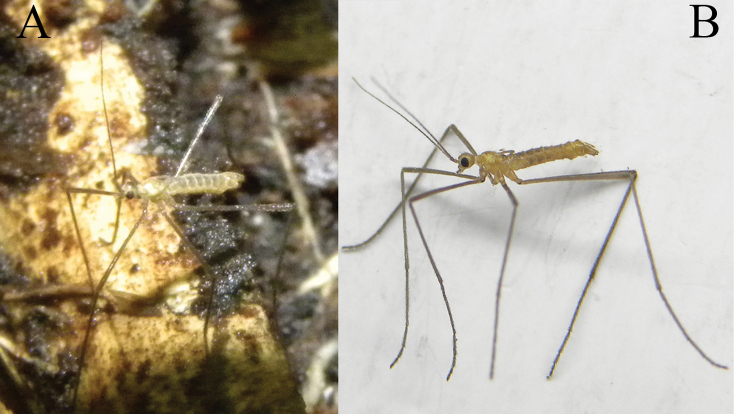
Male Ormosia (Oreophila) komazawai Kato & Kolcsár, sp. nov. **A** habitus, living specimen **B** habitus, freshly killed specimen.

**Figure 7.**
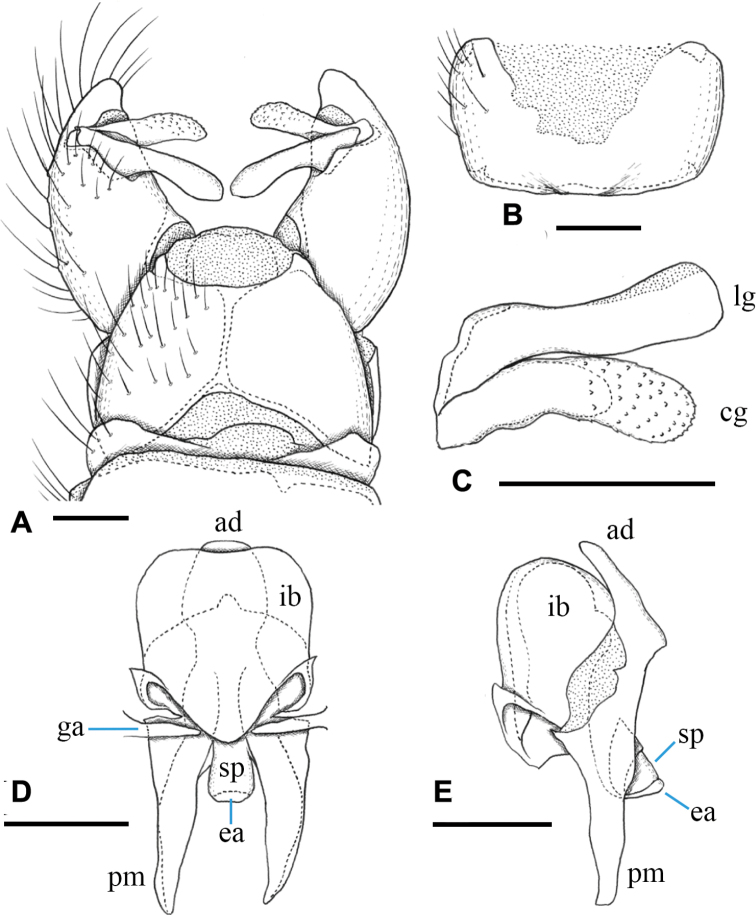
Male terminalia of Ormosia (Oreophila) komazawai Kato & Kolcsár, sp. nov. **A** dorsal view **B** sternite 9, ventral view **C** gonostylus, outer surface **D** aedeagal complex, dorsal view **E** aedeagal complex, lateral view (left = dorsal). Scale bars: 0.1 mm.

Figs 1B , 5A , 6 , 7

##### Type material examined.

***Holotype*.** ♂, pinned. Original label: “Hokkaido, Kucchan-chô-Iwaoto, tributary of Iou-gawa River; alt. 770 m; 42.88333°N, 140.65227°E; 6 Jul. 2015; M. Komazawa leg.” BLKU. “HOLOTYPE Ormosia (Oreophila) komazawai Kato & Kolcsár, sp. nov. [red label]”; BLKU.

***Paratype*.** Japan: [Hokkaido] • 2 ♂; same data as holotype • 2 ♂; same data as holotype except 5 Jul. 2016, BLKU • 1 ♂; same data as previous, except CKLP. • 1 ♂; same data, except 2 July 2022; CMK; M. Komazawa leg.; • 3 ♂ same data as previous, except CKLP; • 6 ♂ same data as previous, except BLKU.

##### Diagnosis.

General coloration yellow (Fig. 1B, 6). Vertex brown medially. Palpus 1-segmented. Antenna often brown on flagellum, very long, 1–1.4× as long as body in male. Wing reduced, ~ 2/3 length of thorax. Legs ochreous, yellowish proximal to basal parts of femora. Male terminalia: tergite 9 bearing pair of small triangular lobes at caudal margin. Gonocoxite distinctly produced beyond base of clasper of gonostylus. Clasper of gonostylus slightly shorter than lobe of gonostylus, almost same width in whole length, ca. as wide as lobe of gonostylus. Interbases fused medially into roundish sac-like plate, without mesal-apical lobe. Female unknown.

##### Description.

**Male.** Body length 1.8–2.2 mm, wing length 0.6 mm.

***Head***: covered with yellow to brown setae. Vertex yellow, widely brown along medial longitudinal line (Fig. 6A), anterior part of vertex roundly convex (Figs 1B, 6B). Eyes small and widely separated, 1/2 as wide as narrowest point of vertex, ~ 1/3 length of head including rostrum in dorsal view. Rostrum yellow, small, ~ 1/4 length of eye in lateral view. Palpus yellow, 1-segmented, roughly bacilliform, dilated distally, 1/4 length of head. Labellum yellow. Antenna very long, 1–1.4× as long as body length (Fig. 6), 15-segmented; scape yellow, 1.5–2× as long as wide; pedicel yellow, oval, 1/2–2/3 length of scape; flagellomeres yellow to brown, slender cylindrical, each segment as long as scape + pedicel or slightly longer, distal segments shorter, apical segment 1/2–2/3 length of flagellomere 1; each flagellomere covered with abundant sensilla, and with 1–3 verticils present only on basal two flagellomeres, at most 1/5 as long as each segment.

***Thorax***: covered with yellow setae. Antepronotum yellow, postpronotum whitish. Mesonotum yellow, dorsoventrally flattened. Prescutal and tuberculate pits indistinct. Pleuron yellow. Wing greatly reduced, ~ 0.6 mm, 5–6× as long as wide, ~ 2/3 length of thorax (Fig. 6B), dusky yellow, paler on basal part, covered with yellow setae. Veins vestigial except one stout vein, probably corresponding to vein R. Halter very slender, weakly dilated on knob, ~ 2/3 length of thorax. Legs with coxae and trochanters yellow, coxae relatively larger than those of non-flightless species; femora ochreous, basal parts more yellowish toward bases; tibiae and tarsi ochreous, sometimes slightly darker on distal one or two segments of tarsi (Figs 1B, 6B).

***Abdomen***: yellow (Fig. 1B), pale brown on living and freshly killed specimens (Fig. 6), densely covered with yellow setae.

***Male terminalia*** (Fig. 7): Tergite 9 with pair of small triangular lobe at caudal margin, less than 1/4 length of middle of tergite 9; anterior margin of tergite 9 deeply and widely notched; tergite 9 slightly wider than long including caudal lobe (Fig. 7A). Sternite 9 largely membranous on posteromedial part (Fig. 7B), anteromedial part convex ventrally in lateral view. Gonocoxite gradually narrowing towards tip, slightly longer than tergite 9, posteroventral margin distinctly and roundly produced beyond base of clasper of gonostylus, produced part ~ 1/2 length of clasper of gonostylus (Fig. 7A). Gonocoxal apodeme short, connected to anterolateral part of interbase (Fig. 7D). Clasper of gonostylus dark and scabrous, slightly shorter than lobe of gonostylus, almost same width in whole length, rounded at tip, weakly curved dorsally on distal part, ca. as wide as lobe of gonostylus in apical view (Fig. 7C). Lobe of gonostylus long finger-shaped, slightly curved, ~ 1/2 length of gonocoxite, distal part flattened (Fig. 7C). Interbases fused medially into roundish sac-like plate, ca. as long as wide in dorsal view, posterior margin almost straight or slightly concave, anterolateral part with short arm (Fig. 7D). Paramere roughly blade-shaped, ca. as long as interbase (Fig. 7D, E). Aedeagus dorsoventrally flattened, ~ 1/3 width of sac-like interbase or slightly wider, weakly constricted near middle in dorsal view, tip slightly beyond apex of interbase (Fig. 7D). Sperm pump angular in dorsal view (Fig. 7D), anterior end situated at basal 1/3 of paramere (Fig. 7D, E). Ejaculatory apodeme poorly developed (Fig. 7D, E).

**Female.** Unknown.

##### Etymology.

This spectacular and unique species is named in honor of its collector, Masaki Komazawa.

##### Habitat and biology.

Masaki Komazawa observed specimens walking on the surface of fallen leaves or on the surface of soil just after snow melt (Fig. 6A).

##### Distribution.

Japan (Hokkaido) (Fig. 5A).

##### Remarks.

The subapterous male of this species is unique in the subgenus, but brachypterous female is known in a Nearctic species, Ormosia (Oreophila) parviala Petersen & Gelhaus, 2004 (Petersen et al. 2004). This species is similar to Ormosia (Oreophila) confluenta in terms of body coloration, Ormosia (Oreophila) longicornis Savchenko, 1980 (Savchenko 1980) from Kazakhstan in terms of long antenna reaching (almost) apex of abdomen if bent backward, and Ormosia (Oreophila) bergrothi (Strobl, 1895) in terms of structure of male terminalia.

#### ﻿ Ormosia (Oreophila) sootryeni

Taxon classificationAnimaliaDipteraLimoniidae

Lackschewitz, 1935

D1945007-2DF4-56BD-BD5F-E42B782F6610

**Figure 8.**
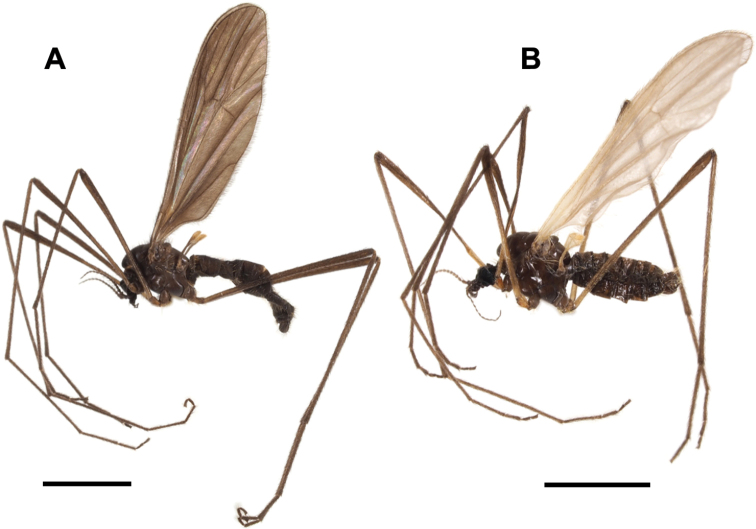
Habitus of male **A**Ormosia (Oreophila) sootryeni Lackschewitz, 1935 **B**Ormosia (Parormosia) nippoalpina Alexander, 1941. Scale bars: 2 mm.

**Figure 9.**
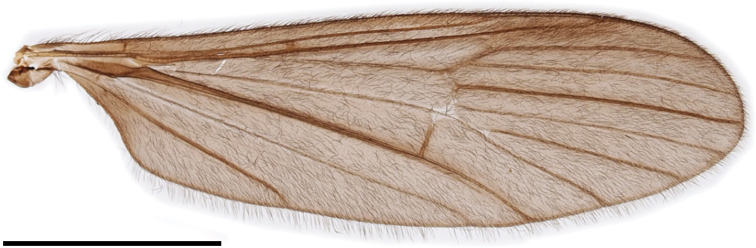
Wing of Ormosia (Oreophila) sootryeni Lackschewitz, 1935. Scale bar: 2 mm.

**Figure 10.**
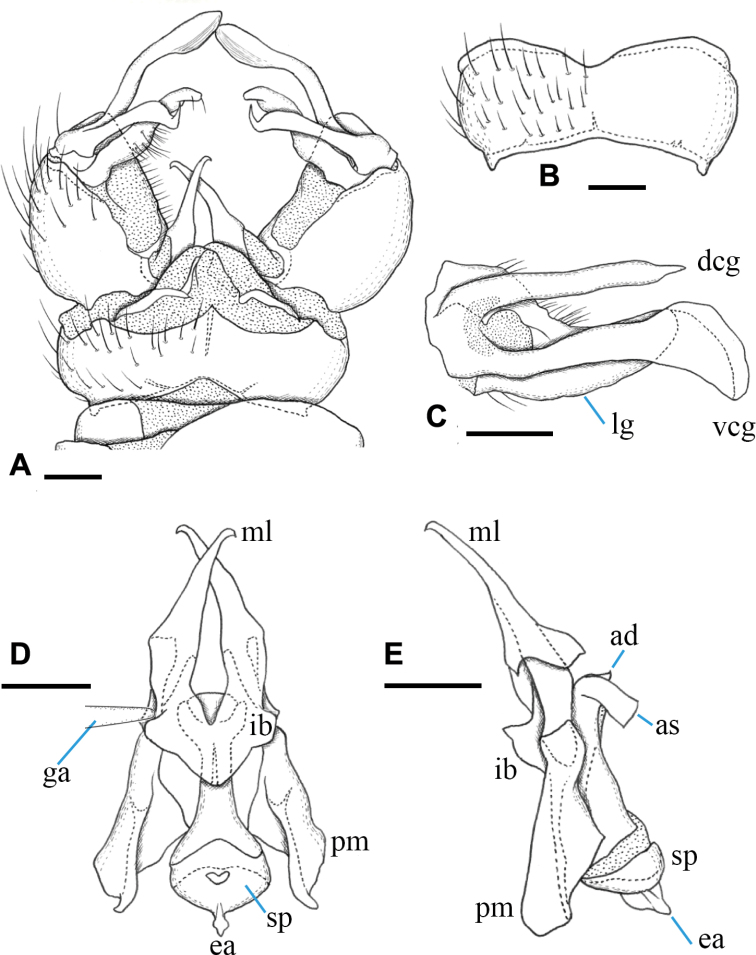
Male terminalia of Ormosia (Oreophila) sootryeni Lackschewitz, 1935 **A** dorsal view **B** sternite 9, ventral view **C** gonostylus, outer surface **D** aedeagal complex, dorsal view (left gonocoxal apodeme omitted) **E** aedeagal complex, lateral view (left = dorsal). Scale bars: 0.1 mm.

**Figure 11.**
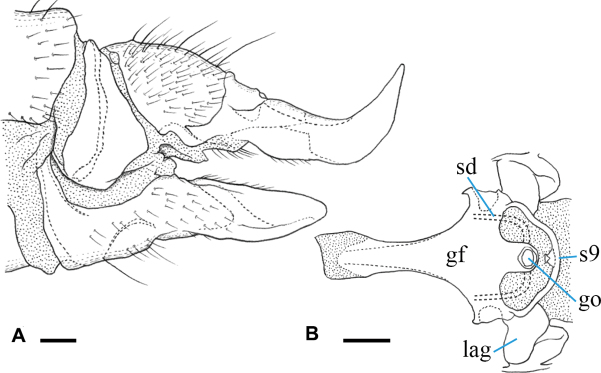
Female terminalia of Ormosia (Oreophila) sootryeni Lackschewitz, 1935 **A** lateral view **B** genital frame, ventral view (left = anterior). Scale bars: 0.1 mm.

Figs 5B , 8A , 9 , 10 , 11

Oreophila sootryeni in Lackschewitz 1935: 8: original description (type locality: Norway, Røsvik).
Ormosia (Ormosia) ducalis in Alexander 1938: 162: original description (type locality: North Korea, Ompo).
Ormosia ducalis in Alexander 1970: 77: faunistic record.
Ormosia (Oreophila) sootryeni in Savchenko 1983: 82: faunistic records, comparison; Nakamura 2014: 32: distribution; Oosterbroek 2022: distribution.

##### Type material examined.

Ormosia (Oreophila) ducalis Alexander, 1938: ***Holotype*** • North Korea, ♂, Ompo; alt. 170 feet; 23 May 1937; A. Yankovsky leg.; USNM.

##### Non-type material examined.

*Ormosiaducalis* Alexander, 1938: Japan • 1 ♂, Hida, On-take; 15 Jul. 1958, Mishima leg.; USNM. North Korea • 1♂; Ompo; alt. 100 feet; 19 May 1938; A. Yankovsky leg.; USNM.

Ormosia (Oreophila) sootryeni Lackschewitz, 1935: Japan • 3 ♂, 1 ♀; Honshu, Aomori, Nishimeya-mura, Kawaratai, Ôkawa-rindô Path; 40.50062°N, 140.20405°E; alt. 300 m; 30 May 2014; • 2 ♂; same locality; 3 Jun. 2014; D. Kato leg.; BLKU. • 1 ♂; Honshu, Aomori, Towada-shi, Okuse, Tsutanuma-rindô Path; 40.590842°N, 140.957052°E; alt. 460 m; 5 Jul. 2014; • 1 ♂; same locality; 30 Aug. 2014; • 1 ♂; same locality; 30 Sep. 2014; D. Kato leg.; BLKU. • 1 ♀; Honshu, Nagano, Sakae-mura, Sakai, Koakazawa-gawa River; 36.85352°N, 138.66358°E; alt. 1310–1500 m; 23 Jul. 2019; • 1 ♂; same locality; 19 Sep. 2019; D. Kato leg.; BLKU.

##### Diagnosis.

General coloration brownish black (Fig. 8A). Vertex often brownish grey. Antenna dark brown. Wing blackish tinged, stigmal region weakly dark. Halter yellow at base and knob. Legs entirely brownish black. Male terminalia: tergite 9 bearing of three small triangular lobes at caudal margin. Gonocoxite slightly produced beyond base of clasper of gonostylus. Clasper of gonostylus divided into two long arms, dorsal arm almost straight, strongly narrow and bent outward at tip, ventral arm slightly longer than dorsal arm, apical part strongly widened and rounded. Interbase with mesal-apical lobe long blade-shaped, curved at extreme tip, basal part of each interbase fused with each other. Female terminalia with cercus stout, strongly upcurved. Hypogynial valve rounded at tip. Genital frame with lateral arm of genital fork roundish, situated at posterior end of genital fork. Sternite 9 slender, arched bridge-shaped.

##### Redescription.

**Male.** Body length 3.8–5.9 mm, wing length 5.7–7.8 mm.

***Head***: covered with black setae. Vertex brownish black, brownish grey by pruinosity from certain angles. Eyes relatively large and widely separated, ~ 4/5 as wide as narrowest point of vertex, ~ 1/2 length of head including rostrum in dorsal view. Rostrum dark brown, ~ 1/3 length of eye in lateral view. Palpus dark brown, 5-segmented, 3/4 length of head, palpomere 1 globular and small, palpomeres 2, 4, and 5 cylindrical, palpomere 3 oval. Labellum dark brown. Antenna dark brown, ~ 3× as long as head, 16-segmented; scape 2× as long as wide; pedicel oval, 1/2 length of scape; flagellomeres bacilliform, gradually slender toward apical segment; each flagellomere with ca. eight verticils, longest one except in apical segment ~ 2.5× as long as each segment.

***Thorax***: covered with black setae on dorsal part and partly with yellow setae on pleuron, coxae, and trochanters. Antepronotum brownish black; postpronotum dusky yellow. Mesonotum subnitidous, brownish black, greyish ochreous by pruinosity from certain angles, weakly yellowish around humeral part. Prescutal pit black, long oval to long bacilliform. Tuberculate pit absent. Pleuron dark brown, slightly variegated with lighter brown. Wing (Fig. 9) tinged with black, stigmal region weakly dark; 3.3–3.5× as long as wide; Sc ending between level of R_2_ and fork of R_2+3+4_; crossvein sc-r distinct, situated between levels of basal 1/7–1/4 of Rs; R_2+3+4_ 1/10 length of R_3_ or shorter; R_2_ situated 1–2× length of itself distal to fork of R_2+3+4_; M_4_ 2–4× as long as M_3+4_; wing margin between tips of CuP and A_1_ 2–3× as long as that between tips of CuP and CuA; A_1_ almost straight. Halter dark brown, base and knob yellow, ~ 2/3 length of thorax. Legs brownish black (Fig. 8A).

***Abdomen*** (Fig. 8A): brownish black, densely covered with black setae mainly on dorsal part and with yellow setae mainly on lateral and ventral parts.

***Male terminalia*** (Fig. 10): Tergite 9 with three small triangular lobes at caudal margin, middle one 2× longer as lateral one; lateral one 1/4 length of middle of tergite 9 including middle lobe; anterior margin of tergite 9 widely notched; tergite 9 approximately 3× wider than long including caudal lobe (Fig. 10A). Sternite 9 widely concave at middle of posterior margin (Fig. 10B). Gonocoxite stout, 2× as long as tergite 9, posteroventral margin weakly and roundly produced beyond base of clasper of gonostylus, produced part less than 1/10 length of clasper of gonostylus (Fig. 10A). Gonocoxal apodeme short, connected to anterolateral part of interbase (Fig. 10D). Clasper of gonostylus dark and smooth on surface, divided into two arms; dorsal arm roughly rod-shaped, almost straight in apical view, slightly shorter than gonocoxite, tip pointed, suddenly narrow and bent outward, claw-like in dorsal view; ventral arm slightly longer than gonocoxite, weakly sinuous, apical part strongly widened and rounded (Fig. 10C). Lobe of gonostylus slightly shorter than gonocoxite, flattened, gradually narrow and curved dorsally toward tip, rounded at tip, middle part 2× as wide as middle part of dorsal arm of clasper of gonostylus (Fig. 10C). Interbases with mesal-apical lobe long blade-shaped, gradually narrow distally, directed posterodorsally, curved at extreme tip, basal part fused with each other, anterolateral part roundly produced laterally (Fig. 10D). Paramere wide, roughly triangular, ca. as long as interbase (Fig. 10D). Aedeagus relatively flat, distinctly broad at tip in dorsal view (Fig. 10D), apical part ~ 1/3 width of basal part of interbase, tip slightly beyond furcation point of interbase (Fig. 10D), with short extension of aedeagal sheath directed ventrally (Fig. 10E). Sperm pump roundish in dorsal view, anterior end situated at level of anterior end of paramere (Fig. 10D). Ejaculatory apodeme developed, laterally compressed, fin-like plate, ~ 1/3 length of diameter of sperm pump (Fig. 10E).

**Female.** Body length 6.0–6.4 mm, wing length 7.4–8.1 mm. Generally resembling male.

***Female terminalia*** (Fig. 11): brownish black, distal part of tergite 10 yellowish; cercus amber-colored, basal 1/2 dark; hypogynial valve dusky yellow, base of lateral part brownish. Tergites 8 and 9 fused. Cercus stout, strongly upcurved, slightly shorter than tergite 10; hypogynial valve ca. as long as sternite 8, finger-shaped in lateral view, rounded at tip, basal part ca. as wide as that of cercus, tip ending near level of middle of cercus (Fig. 11A). Genital frame with genital fork cross-shaped, extended laterally at posterior 1/4, weakly constricted medially, anterolateral corner of lateral extension pointed; lateral arm of genital fork roughly roundish, situated at posterior of genital fork; sternite 9 slender, arched bridge-shaped, arising from posterolateral corner of genital fork, middle part with small lobe (Fig. 11B). Two spermathecal ducts present, spermathecae indistinct.

##### Distribution.

Japan (Honshu) (Fig. 5B), North Korea, Russia (FE), Kuril Islands, Kazakhstan (east), Finland, Norway, and Sweden.

##### Remarks.

This species is similar to a Chinese species, Ormosia (Oreophila) subducalis Alexander, 1940 (Alexander 1940b), but is differentiated from it by the following characters: wing entirely blackish tinged (brownish yellow with clearer yellow base in Ormosia (Oreophila) subducalis); halter dark brown, base and knob yellow (orange yellow in Ormosia (Oreophila) subducalis).

#### ﻿ Subgenus Parormosia

Taxon classificationAnimaliaDipteraLimoniidae

Alexander, 1965

63ACBD4E-0B6F-5094-822D-E299A03091DF

##### Type species.

*Rypholophusnigripilus* Osten Sacken, 1869 (Osten Sacken 1869) by original designation.

##### Note.

This subgenus includes 23 species, before this article (5 Palaearctic, 9 Nearctic, and 9 Oriental species) (Oosterbroek 2022).

#### ﻿ Ormosia (Parormosia) diversipes

Taxon classificationAnimaliaDipteraLimoniidae

Alexander, 1919

048E7270-F88C-500A-8B07-E55B46B87C6B

**Figure 12.**
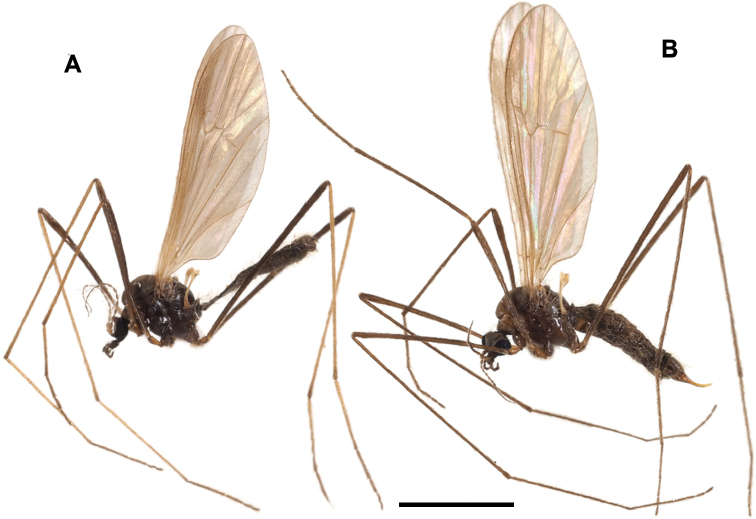
Habitus of Ormosia (Parormosia) diversipes Alexander, 1919. **A** male **B** female. Scale bar: 2 mm.

**Figure 13.**
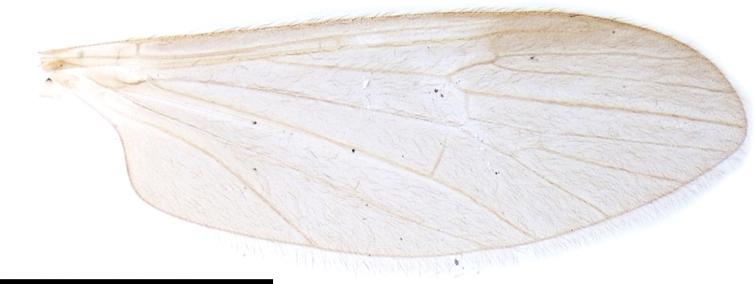
Wing of Ormosia (Parormosia) diversipes Alexander, 1919. Scale bar: 2 mm.

**Figure 14.**
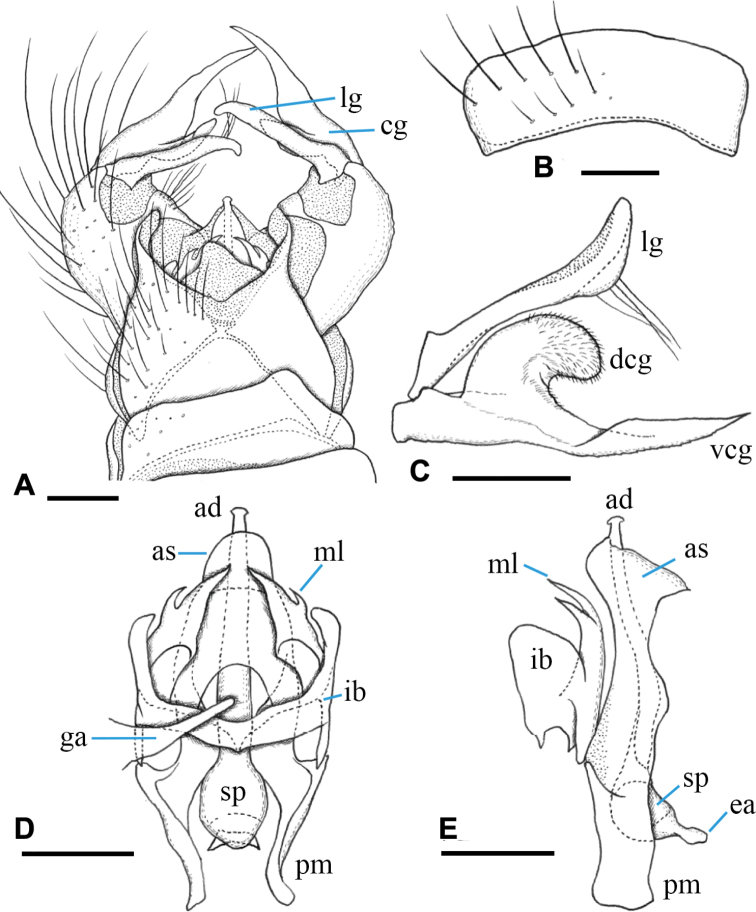
Male terminalia of Ormosia (Parormosia) diversipes Alexander, 1919 **A** dorsal view **B** sternite 9, ventral view **C** gonostylus, outer surface **D** aedeagal complex, dorsal view (tip of gonocoxal apodeme moved posteriorly and left gonocoxal apodeme omitted) **E** aedeagal complex, lateral view (left = dorsal). Scale bars: 0.1 mm.

**Figure 15.**
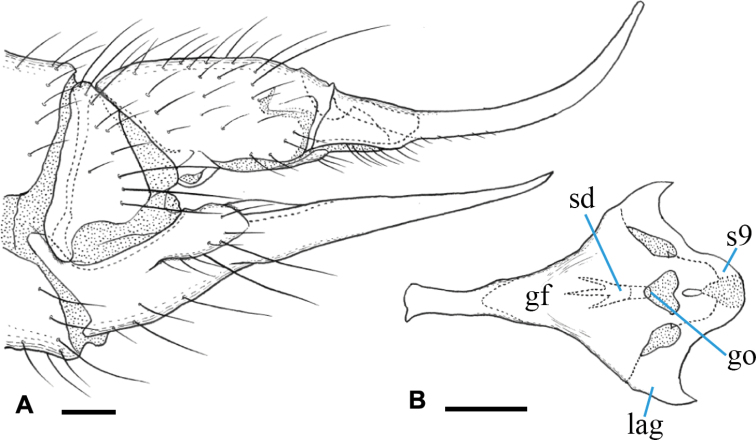
Female terminalia of Ormosia (Parormosia) diversipes Alexander, 1919 **A** lateral view **B** genital frame, ventral view (left = anterior). Scale bars: 0.1 mm.

Figs 5C , 12 , 13 , 14 , 15

Ormosia diversipes in Alexander 1919: 334: original description (type locality: Japan, Honshu, Saitama, Chichibu); Alexander 1930: 508: faunistic record; Alexander 1936a: 193: comparison.
Ormosia atripes in Alexander 1919: 335: original description (type locality: Japan, Honshu, Tokyo, Meguro); Alexander 1936a: 193: suggestion of synonymy; Alexander 1965: 35: synonymy.
Ormosia (Ormosia) atripes in Alexander 1953b: 173: faunistic record; Alexander 1954: 31: faunistic record, distribution.
Ormosia (Parormosia) diversipes in Alexander 1965: 35: new subgeneric combination; Savchenko and Krivolutskaya 1976: 94: faunistic record; Savchenko 1979: 27: faunistic record; Savchenko 1983: 83: faunistic records; Pilipenko and Sidorenko 2006: distribution; Pilipenko 2009: 333: faunistic record; Nakamura 2014: 33: distribution; Oosterbroek 2022: distribution.

##### Type material examined.

*Ormosiaatripes* Alexander, 1919: ***Paratype*.** Japan • 1 ♀; Honshu; Tokio (Tokyo), Meguro; 9 Apr. 1919; R. Takahashi leg.; USNM.

*Ormosiadiversipes* Alexander, 1919: ***Paratype*.** Japan • 1 ♂; Honshu; Tokio (Tokyo), Meguro; 26 Mar. 1919; R. Takahashi leg.; USNM.

##### Non-type material examined.

Ormosia (Parormosia) diversipes Alexander, 1919: Japan • 1 ♂; Honshu, Aomori, Fukaura-machi, Okazaki, Fukaura House; 40.63684°N, 139.9116°E; alt. 130 m; 26 Aug. 2014; light trap; D. Kato leg.; BLKU. • 1 ♂; Honshu, Aomori, Fukaura-machi, Mt. Takaniô-yama; alt. 140 m; 11 May 2014; D. Kato leg.; BLKU. • 1 ♂; Honshu, Aomori, Hirosaki-shi, Koguriyama, Inekari-sawa River; 40.53658°N, 140.48701°E; alt. 170 m; 10 May 2013; • 1 ♂; same locality; 17 May 2013; D. Kato leg.; BLKU. • 1 ♂; Honshu, Aomori, Hirosaki-shi, Ichinowatari-Washinosu; 40.51923°N, 140.43889°E; alt. 205 m; 9 May 2013; • 1 ♂; same locality; 12 May 2013; • 1 ♂; same locality; 21 May 2013; • 1 ♂; same locality; 5 Sep. 2013; D. Kato leg.; BLKU. • 1 ♀; Honshu, Aomori, Nakadomari-machi, Ôsawanai, Ôsawanai-tameike Pond; 40.9464°N, 140.4623°E; alt. 35 m; 15 May 2014; • 1 ♂; same locality; 24 May 2014; D. Kato leg.; BLKU. • 1 ♂, 1 ♀; Honshu, Aomori, Nishimeya-mura, Kawaratai, Ôkawa-rindô Path; 40.50062°N, 140.20405°E; alt. 300 m; 18 Sep. 2013; • 1 ♂; same locality; 13 May 2014; D. Kato leg.; BLKU. • 1 ♀; Honshu, Aomori, Nishimeya-mura, Kawaratai, The Shirakami Natural Science Park, Hirosaki Univ.; 40.5188°N, 140.21488°E; alt. 255 m; 18 May 2014; • 2 ♂; same locality; 25 May 2014; • 2 ♂; same locality; 1 Jun. 2014; • 1 ♂; same locality; 28 Sep. 2014; light trap; D. Kato leg.; BLKU. • 2 ♂, 4 ♀; Honshu, Aomori, Towada-shi, Okuse, Tsutanuma-rindô Path; 40.59084°N, 140.95705°E; alt. 460 m; 14 May 2014; D. Kato leg.; BLKU. • 1 ♂; Honshu, Niigata, Tôkamachi-shi, Matsunoyama-Amamizukoshi, Mt. Amamizu-yama; alt. 920 m; 1 Oct. 2019; D. Kato leg.; BLKU. • 2 ♂; Honshu, Niigata, Tôkamachi-shi, Matsunoyama, Echigo-Matsunoyama Museum of Natural Science ‘Kyororo’; 37.09956°N, 138.61631°E; alt. 310 m; 25 Apr. 2020; • 1 ♀; same locality; 5 May 2020; D. Kato leg.; BLKU. • 1 ♂; Honshu, Niigata, Tôkamachi-shi, Matsunoyama-Mizunashi, Step-in-plan; alt. 230 m; 2 Oct. 2020; D. Kato leg.; BLKU. • 1 ♂; Honshu, Nagano, Sakae-mura, Sakai, Koakazawa-gawa River; 36.85352°N, 138.66358°E; alt. 1320–1400 m; 21 Aug. 2020; D. Kato leg.; BLKU. • 1 ♂; Honshu, Gifu; 15 Nov. 1931; S. Kariya leg.; USNM. • 1 ♂; Honshu, Hida, Ontake; 26 Jul. 1959; Mishima leg.; USNM. • 1 ♀; Honshu, Ibaraki, Tsukuba-shi, Oda, Mt. Hôkyô-san; 36.15802°N, 140.12142°E; alt. 50 m; 14 Oct. 2011; • 1 ♂; same locality; 26 Mar. 2013; D. Kato leg.; BLKU. • 1 ♂; Honshu, Ôsaka, Izumisano-shi, Ôgi, Inunakisan Spa; 34.3398°N, 135.38375°E; alt. 250 m; 16 Apr. 2014; D. Kato leg.; BLKU. • 2 ♂; Honshu, Okayama, Okayama, Maniwa-shi, Hiruzen-Shimotokuyama; 35.3293°N, 133.59725°E; alt. 780 m; 17 May 2015; D. Kato leg.; BLKU. • 3 ♀; Shikoku, Kagawa, Mannȏ-chȏ, Katsuura, Myȏjin-gawa River; 34.09402°N, 134.0143°E; alt. 480 m; 21 Apr. 2014; D. Kato leg.; BLKU. • 1 ♂; Shikoku, Ehime, Kumakogen, Kuma River, dam; 33.68392°N, 132.87145°E; alt. 615 m; 10 Oct. 2021; L.-P. Kolcsár leg.; CKLP. • 2 ♂, 1 ♀; Shikoku, Ehime, Kumakogen, Omogo River; 33.56118°N, 133.00669°E; alt. 290 m; 13 Oct. 2021; light trap; L.-P. Kolcsár leg.; CKLP. • 1 ♂; Shikoku, Ehime, Kumakogen, Myogadani River; 33.54869°N, 132.94807°E; alt. 890 m; 17 Jun. 2019; L.-P. Kolcsár leg.; CKLP. • 1 ♂; Shikoku, Ehime, Kumakogen, small stream and concrete wall; 33.59423°N, 132.98385°E; alt. 640 m; 19 May 2019; L.-P. Kolcsár leg.; CKLP. • 1 ♂; Shikoku, Ehime, Matsuyama, bamboo forest and small stream; 33.86363°N, 132.76673°E; alt. 110 m; 24 Mar. 2021; L.-P. Kolcsár leg.; CKLP. • 1 ♂; Shikoku, Ehime, Matsuyama, fill up lake; 33.83858°N, 132.81399°E; alt. 100 m; 27 Apr-. 2019; L.-P. Kolcsár leg.; CKLP. • 1 ♂, 1 ♀; Shikoku, Ehime, Matsuyama, stream; 33.86152°N, 132.82591°E; alt. 180 m; 5 Apr. 2019; L.-P. Kolcsár leg.; CKLP. • 1 ♂; Shikoku, Ehime, Matsuyama, rocky stream and waterfall; 33.86801°N, 132.83482°E; alt. 240 m; 3 May 2019; L.-P. Kolcsár leg.; CKLP. • 1 ♂; Shikoku, Ehime, Matsuyama, small ruderal stream; 33.863284°N, 132.771579°E; alt. 125 m; 10 Apr. 2020; • 7 ♂, 1 ♀; Shikoku, Ehime, Matsuyama, Takimoto, small lake; 33.96114°N, 132.82899°E; alt. 205 m; 10 Oct. 2021; light trap; L.-P. Kolcsár leg.; CKLP. • 8 ♂, 4 ♀; Shikoku, Ehime, Matsuyama, Tateiwa dam; 33.97129°N, 132.87542°E; alt. 310 m; 10 Oct. 2021; light trap; L.-P. Kolcsár leg.; CKLP. • 1 ♂; Shikoku, Ehime, Matsuyama, small waterfall in Japanese cedar forest; 33.85911°N, 132.83472°E; alt. 330 m; 17 May 2020; L.-P. Kolcsár leg.; CKLP. • 1 ♂; Shikoku, Ehime, Seiyo, Shirokawachō Noigawa; 33.44036°N, 132.81202°E; alt. 600 m; 11 May 2021; L.-P. Kolcsár leg.; CKLP. • 5 ♂ (1 ♂: BOLD ID: JPCOI003-22), 2 ♀ (1♀: BOLD ID: JPCOI004-22); Shikoku, Ehime, Toon, Kamihayashi Forest Park; 33.72146°N, 132.89012°E; alt. 1100 m; light trap; 3 Oct. 2021. • 2 ♀; Shikoku, Tokushima, Higashimiyoshi-chô, Higashiyama, Ogawadani-gawa River; alt. 340 m; 21 Apr. 2014; D. Kato leg.; BLKU. • 1 ♀; Shikoku, Tokushima, Miyoshi-shi, Higashiiya-Ochiai, near Matsuogawa Dam; 33.96478°N, 133.93908°E; alt. 900 m; 15 May 2015; • 1 ♂; same locality; 30 Apr. 2016; D. Kato leg.; BLKU. • 1 ♂; Shikoku, Tokushima, Miyoshi, Higashiiya, Mt. Tsurugi area; 33.86651°N, 134.08549°E; alt. 1315 m; light trap; 16 Oct. 2021; K. Kuroda leg.; CKLP. • 1 ♂; Kyushu, Fukuoka, Fukuoka-shi, Jônan-ku, Katae, Mt. Abura-yama; 33.53046°N, 130.36594°E; alt. 220 m; 19 Apr. 2014; • 1 ♂, 1 ♀; same locality; 20 Oct. 2015; D. Kato leg.; BLKU. • 2 ♂; Kyushu, Fukuoka, Fukuoka-shi, Sawara-ku, Itaya, Mt. Sefuri-san; 33.43811°N, 130.36673°E; alt. 970 m; 23 May 2015; • 1 ♂, 2 ♀; same locality; 10 Jun. 2015; • 2 ♂; same locality; 4 Oct. 2015; D. Kato leg.; BLKU. • 1 ♀; Kyushu, Fukuoka, Itoshima-shi, Shimasakurai, Ôguchi seaside; 33.63249°N, 130.18094°E; alt. 5 m; 23 Oct. 2015; D. Kato leg.; BLKU. • 2 ♀; Kyushu, Fukuoka, Miyawaka-shi, Inunaki, Mt. Inunaki-san; 33.68112°N, 130.55317°E; alt. 300 m; 5 May 2015; D. Kato leg.; BLKU. • 1 ♀; Kyushu, Kumamoto, Yatsushiro-shi, Izumi-machi-Hagi, Momiki-gawa River; 32.51417°N, 130.93927°E; alt. 580 m; 11 May 2016; D. Kato leg.; BLKU. • 1 ♂; Kyushu, Kumamoto, Yatsushiro-shi, Izumi-machi-Momiki; alt. 1060 m; 22 Sep. 2015; • 1 ♂; same locality; alt. 1400 m; 17 Oct. 2015; D. Kato leg.; BLKU. • 1 ♀; Kyushu, Miyazaki, Gokase-chô, Kuraoka, Gokase ski area; alt. 1500 m; 28 Jun. 2015; D. Kato leg.; BLKU. • 1 ♂; Kyushu, Mt. Kirishima; alt. 2500 ft.; 3 May 1929; S. Issiki leg.; USNM. • 1 ♂; Kyushu, Saga, Karatsu-shi, Hamatama-machi-Torisu, Tsubakiyama-tameike Pond; 33.40414°N, 130.10641°E; alt. 630 m; 26 Apr. 2015; D. Kato leg.; BLKU. • 1 ♂; Kyushu, Saga, Saga-shi, Fuji-machi-Seiya, Kase-gawa River near Hokuzan Dam; 33.43322°N, 130.23212°E; alt. 320 m; 23 Apr. 2015; D. Kato leg.; BLKU. • 1 ♀; Kyushu, Kagoshima, Yakushima I, Mugio, close to Yakusugi Land; 30.30173°N, 130.58574°E; alt. 1100 m; 19 Oct. 2021; L.-P. Kolcsár leg.; CKLP. • 1 ♂; Kyushu, Kagoshima, Yakushima I, Nagata, Nagata River; 30.38757°N, 130.43528°E; alt. 40 m; 20 Oct. 2021; L.-P. Kolcsár leg.; CKLP.

##### Diagnosis.

General coloration brown to dark brown (Fig. 12). Vertex greyish. Antenna yellowish on pedicel and at least basal segments of flagellum. Mesonotum sometimes barely greyish. Wing brownish tinged, stigmal region weakly dark. Cell dm usually closed. Halter dusky yellow. Legs dark brown in female (Fig. 12B), tibiae to tarsi mostly yellow in male (Fig. 12A). Male terminalia: tergite 9 bearing pair of long membranous lobes at caudal margin. Clasper of gonostylus divided into two arms near base, ventral arm blade-shaped, gradually narrow toward tip, ~ 2× as long as curved stout dorsal arm. Lobe of gonostylus narrower and slightly shorter than clasper, weakly dilated at apical 1/4, with long setae on ventral margin near tip. Interbase with mesal-apical lobe bearing two claws. Female terminalia with cercus slender, upcurved distally. Lateral arm of genital fork pointed and curved posteriorly. Sternite 9 rounded posteriorly, desclerotized medially.

##### Redescription.

**Male.** Body length 3.2–4.5 mm, wing length 3.9–5.5 mm.

***Head***: covered with black setae. Vertex grey to dark grey. Eyes relatively large and widely separated, ~ 4/5 as wide as narrowest point of vertex, ~ 1/2 length of head including rostrum in dorsal view. Rostrum dark brown, ~ 1/2 length of eye in lateral view. Palpus dark brown, 5-segmented, slightly shorter than head, palpomere 1 globular and small, palpomeres 2–5 cylindrical, slenderer in palpomeres 2 and 5. Labellum dark brown. Antenna 16-segmented, 2.5–3× as long as head (Fig. 12A); scape dark brown, 1.5× as long as wide; pedicel dusky yellow, roughly globular, 3/4 length of scape; flagellomeres dusky yellow, sometimes darker on distal segments, oval in flagellomere 1 and slender oval on succeeding segments; each flagellomere with one or two verticils, longest one ~ 2× as long as each segment, gradually shorter toward distal segment, sensilla abundant, at most as long as each flagellomere.

***Thorax***: covered with yellow setae, rarely brownish. Pronotum brown to dark brown, often yellowish on lateral side of postpronotum. Mesonotum brown to dark greyish brown, sometimes lighter brown around prescutal pit. Prescutal pit brown to black, oval to long bacilliform, often widened toward outer end. Tuberculate pit distinct, situated at anterior 2/5 between anterior margin of mesonotum and prescutal pit. Pleuron subnitidous, dark brown. Wing (Fig. 13) relatively wide, 3.1–3.2× as long as wide; tinged with brown, sometimes weakly yellowish on prearcular region, stigmal region weakly dark; Sc ending at level of R_2_ or slightly distal to it; crossvein sc-r distinct, situated between levels of basal 1/3–1/2 of Rs; R_2+3+4_ 1/7–1/3 length of R_3_; R_2_ situated between fork of R_2+3+4_ and length of itself distal to it; M_4_ 1–2× as long as M_3+4_; cell dm closed, 0.4–0.7× as long as cell m_1+2_, or sometimes open by atrophy of crossvein m-m; wing margin between tips of CuP and A_1_ 2.5–3× as long as that between tips of CuP and CuA; A_1_ curved posteriorly near middle. Halter dusky yellow, ~ 3/5 length of thorax (Fig. 12A). Legs with coxae to femora dark brown, femora very narrowly pale at bases; tibiae to tarsi yellow to dusky yellow, tips of tibiae and distal segments of tarsi weakly dark (Fig. 12A).

***Abdomen***: dark brown, densely covered with yellow setae.

***Male terminalia*** (Fig. 14): Tergite 9 with pair of long and flat, largely membranous lobes at caudal margin, ~ 1/3 length of remainder of tergite 9; tergite 9 slightly longer than wide including caudal lobe (Fig. 14A). Sternite 9 slightly and widely convex at posterior margin (Fig. 14B). Gonocoxite roundish, slightly shorter than tergite 9, posteroventral margin not produced beyond base of clasper of gonostylus. Gonocoxal apodeme long, connected to each other, forming bridge, central part jointed with anteromedial part of interbase (Fig. 14D). Clasper of gonostylus dark, slightly longer than gonocoxite, divided into two arms; dorsal arm short, stout and curved ventrally, rounded at tip, distal 1/2 densely covered with black microscopic setae; ventral arm ~ 2× as long as dorsal arm, blade-shaped, weakly twisted, gradually narrow toward tip, acute at tip (Fig. 14C). Lobe of gonostylus slender, narrower, and slightly shorter than clasper, weakly dilated at apical 1/4, with several long setae on ventral margin near tip, obtuse at tip (Fig. 14C). Interbase with mesal-apical lobe claw-shaped, curved and directed posterodorsally, bearing smaller spine at middle of outer margin (Fig. 14D, E); basal part of each interbase fused and dorsolateral part roundly produced in lateral view (Fig. 14E). Paramere wide, shorter than interbase (Fig. 14E). Aedeagus slender and cylindrical, extreme tip slightly widened, extending beyond tip of interbase (Fig. 14D, E). Aedeagal sheath covering aedeagus except apical part, posterior end ~ 4× as wide as aedeagus at this point (Fig. 14D), strongly produced ventrally (Fig. 14E). Sperm pump roundish in dorsal view, anterior end situated at level of middle of paramere (Fig. 14D). Ejaculatory apodeme developed, dorsoventrally compressed, fan-like plate, ~ 1/2 length of diameter of sperm pump (Fig. 14D, E).

**Female.** Body length 3.6–5.0 mm, wing length 4.2–5.9 mm. Generally resembling male (Fig. 12B), except antenna shorter, ~ 2× as long as head; flagellomeres often dark brown entirely, with ca. eight verticils on each of basal segments, fewer on each of distal segments, longest one at most 1.5× as long as each segment. Tibiae to tarsi dark brown, concolorous with femora (Fig. 12B).

***Female terminalia*** (Fig. 15): dark brown, cercus turning to amber-color toward tip. Tergites 8 and 9 fused. Cercus slender, upcurved distally, 1.3× longer than tergite 10; hypogynial valve slender, 1.5× as long as sternite 8, gradually narrowed toward tip, basal part ca. as wide as that of cercus, tip ending at level of basal 2/3 of cercus (Fig. 15A). Genital frame with genital fork widened posteriorly, anterior part slender; lateral arm of genital fork pointed at tip and curved posteriorly, situated at posterior end of genital fork; sternite 9 rounded posteriorly, middle part desclerotized; three membranous areas present at level of genital opening (Fig. 15B). Three spermathecal ducts present, basal parts blackened (Fig. 15B). Spermathecae indistinct.

##### Distribution.

Japan (Honshu, Shikoku, Kyushu) (Fig. 5C), Russia (FE), and Kuril Islands.

##### Remarks.

This species resembles a Chinese species, Ormosia (Parormosia) nigripennis Alexander, 1936 (Alexander 1936a), but this species has two-spined mesal-apical lobe of the interbase (Fig. 14D, E), while the latter species has a single pointed lobe on the interbase.

#### ﻿ Ormosia (Parormosia) nippoalpina

Taxon classificationAnimaliaDipteraLimoniidae

Alexander, 1941

B124AF68-C943-5EC4-889F-B37D580B634B

**Figure 16.**
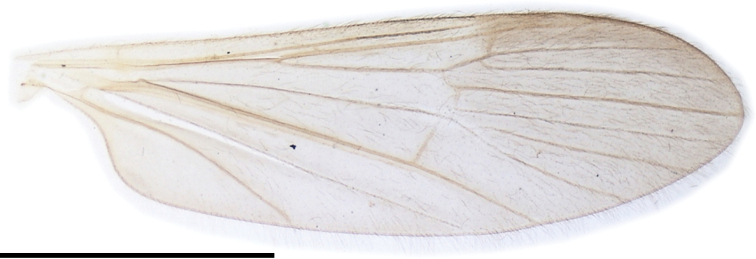
Wing of Ormosia (Parormosia) nippoalpina Alexander, 1941. Scale bar: 2 mm.

**Figure 17.**
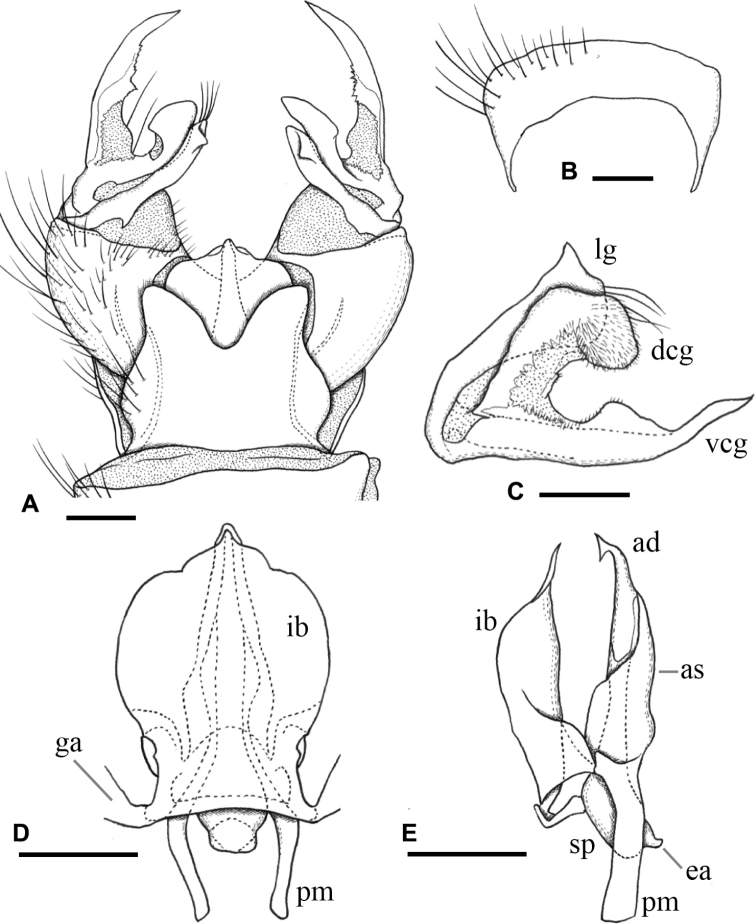
Male terminalia of Ormosia (Parormosia) nippoalpina Alexander, 1941 **A** dorsal view **B** sternite 9, ventral view **C** gonostylus, outer surface **D** aedeagal complex, dorsal view **E** aedeagal complex, lateral view (left = dorsal). Scale bars: 0.1 mm.

**Figure 18.**
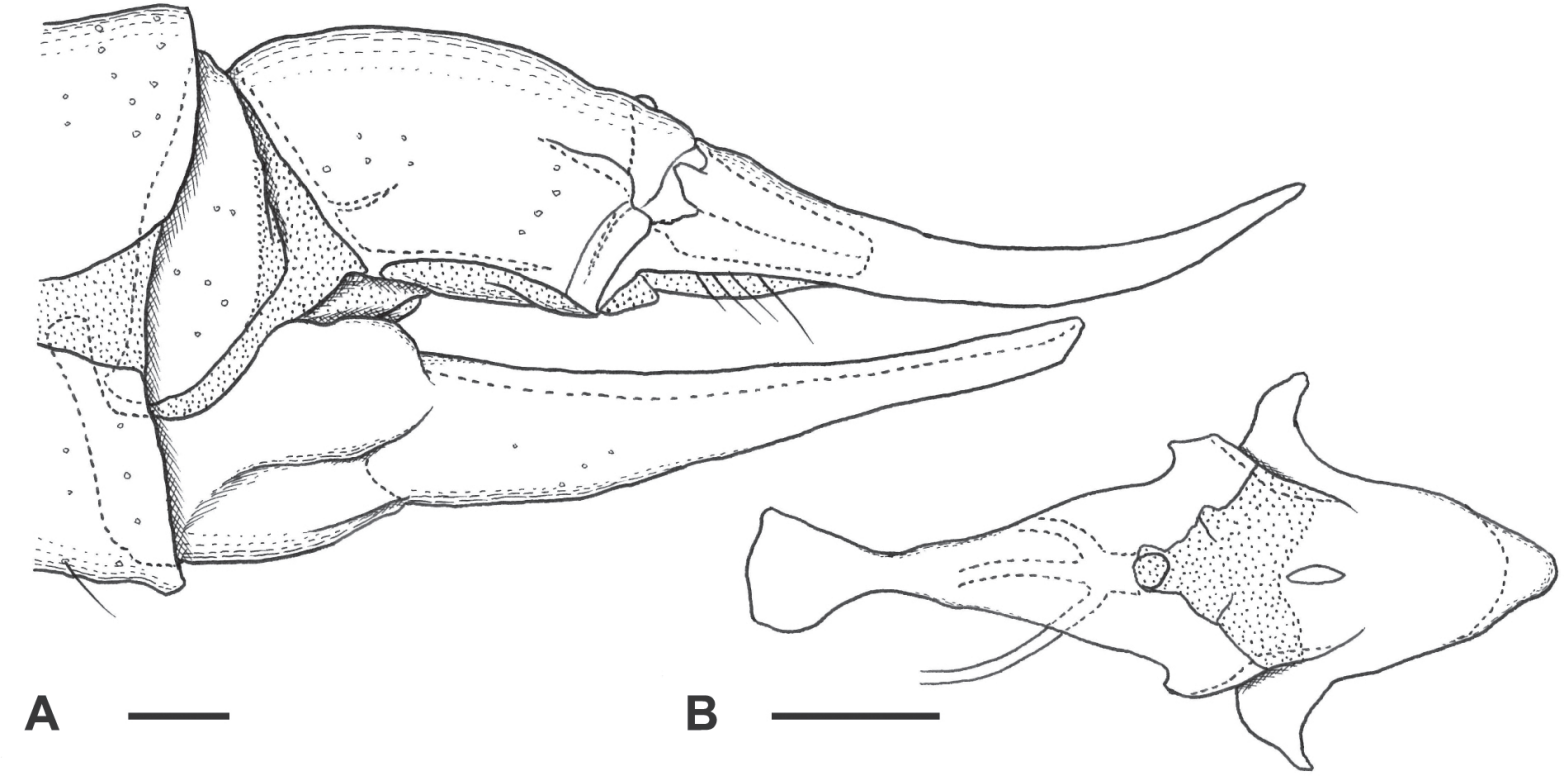
Female terminalia of Ormosia (Parormosia) nippoalpina Alexander, 1941 **A** lateral view **B** genital frame, ventral view (left = anterior). Scale bars: 0.1 mm.

Figs 5D , 8B , 16 , 17 , 18

Ormosia (Ormosia) nippoalpina in Alexander 1941: 63: original description (type locality: Japan, Honshu, Nagano, Kamikochi).
Ormosia (Parormosia) nippoalpina in Savchenko 1979: 27: faunistic records; Nakamura 2014: 33: distribution; Oosterbroek 2022: distribution.

##### Type material examined.

***Holotype*.** Japan • ♂; Honshu, Kamikochi, alt. 5000 feet; 23 Jun. 1939; E. Suenson leg.; USNM.

***Paratype*.** Japan • 1 ♀; same locality and date as holotype; USNM.

##### Non-type material examined.

Japan • 1 ♂; Honshu, Aomori, Nishimeya-mura, Kawaratai, Ôkawa-rindô Path; 40.50062°N, 140.20405°E; alt. 300 m; 13 May 2014. • 1 ♂; same locality; 20 May 2014; D. Kato leg.; BLKU. • 1 ♀; Niigta, Itoigawa-shi, Ôtokoro, near Udo-gawa River; alt. 1128 m; 36.84198°N, 137.82252°E; 14 June 2022; L.-P. Kolcsár leg.; CKLP.

##### Diagnosis.

General coloration brown to dark brown (Fig. 8B). Vertex greyish. Antenna brown to dark brown. Mesonotum subnitidous. Wing brownish tinged, stigmal region barely dark. Cell dm open. Halter dusky yellow. Legs mostly dark brown, yellowish on trochanters and bases of femora. Male terminalia: tergite 9 bearing pair of large triangular lobes at caudal margin. Clasper of gonostylus divided into two arms; ventral arm ~ 1.5× as long as curved stout dorsal arm, roughly blade-shaped, wide at middle and narrow on distal part. Lobe of gonostylus flattened, slightly shorter than clasper, gradually widened distally, widest part ~ 1/3 length of lobe of gonostylus, pointed at apicodorsal corner, distal margin with several long setae. Interbases fused medially into large roundish plate, pointed at tip, mesal-apical lobe absent. Female terminalia with cercus relatively slender, upcurved distally. Lateral arm of genital fork pointed and curved posteriorly. Sternite 9 subacute at posterior end.

##### Redescription.

**Male.** Body length 3.3–4.0 mm, wing length 4.8–5.8 mm.

***Head***: covered with yellow and black setae. Vertex grey to dark grey. Eyes relatively large and widely separated, ~ 3/4 as wide as narrowest point of vertex, ~ 1/2 length of head including rostrum in dorsal view. Rostrum dark brown, ~ 1/2 length of eye in lateral view. Palpus dark brown, 5-segmented, slightly shorter than head, palpomere 1 globular and small, palpomeres 2–5 cylindrical, slenderer in palpomeres 2 and 5. Labellum dark brown. Antenna 16-segmented, 2.5× as long as head; scape dark brown, 2× as long as wide; pedicel brown, roughly globular, 3/4 length of scape; flagellomeres brown, subglobular on basal segments, longer oval on distal segments; each flagellomere with two verticils, longest one ~ 2× as long as each segment, gradually shorter toward distal segment, sensilla abundant, at most 1/3 as long as each flagellomere.

***Thorax***: covered with yellow setae. Pronotum dark brown, yellowish on postpronotum. Mesonotum subnitidous, brown to dark brown, with small yellowish area just above lateral end of postpronotum. Prescutal pit dark brown, oval to long bacilliform. Tuberculate pit distinct, situated at anterior 1/3 to 1/2 between anterior margin of mesonotum and prescutal pit. Pleuron subnitidous, dark brown, weakly yellowish on dorsal part of anepisternum. Wing (Fig. 16) tinged with brown, prearcular region more yellowish, stigmal region barely dark; 3.3–3.4× as long as wide; Sc ending at level of R_2_ or slightly distal to it; crossvein sc-r distinct, situated between levels of basal 1/5–2/5 of Rs; R_2+3+4_ 1/9–1/6 length of R_3_; R_2_ situated between 1/3–1× lengths of itself distal to fork of R_2+3+4_; M_4_ 3.5–10× as long as M_3+4_; cell dm open by atrophy of crossvein m-m; wing margin between tips of CuP and A_1_ 2–2.5× as long as that between tips of CuP and CuA; A_1_ curved posteriorly near middle. Halter yellow, slightly brownish at base, ~ 3/5 length of thorax. Legs with coxae dark brown; trochanters yellow to dusky yellow; femora to tarsi dark brown, basal parts of femora yellowish toward bases (Fig. 8B).

***Abdomen***: dark brown, densely covered with yellow setae.

***Male terminalia*** (Fig. 17): Tergite 9 with pair of large triangular lobes at caudal margin, ~ 1/2 length of remainder of tergite 9; tergite 9 including caudal lobe slightly wider than long (Fig. 17A). Sternite 9 slightly and widely convex at posterior margin, anterior corner distinctly produced anteriorly (Fig. 17B). Gonocoxite roundish, slightly longer than tergite 9, posteroventral margin not produced beyond base of clasper of gonostylus (Fig. 17A). Gonocoxal apodeme short, connected to anterolateral part of interbase (Fig. 17D). Clasper of gonostylus dark, slightly longer than gonocoxite, divided into two arms; dorsal arm stout and curved ventrally in apical view (Fig. 17C), rounded at tip, tip directed distally (Fig. 17A), distal part densely covered with black microscopic setae; ventral arm ~ 1.5× as long as dorsal arm, roughly blade-shaped, wide at middle and narrow on distal part, acute at tip (Fig. 17C). Lobe of gonostylus flattened, slightly shorter than clasper, gradually broadened distally, widest part ~ 1/3 length of lobe of gonostylus, dorsal-apical corner pointed and ventral-apical margin rounded, distal margin with several long setae (Fig. 17C). Interbases fused medially into large roundish plate, almost straight at anterior margin and pointed at posterior margin, basal 1/4 constricted; mesal-apical lobe absent (Fig. 17A, D). Paramere narrow rod-shaped, shorter than interbase (Fig. 17D, E). Aedeagus slender and cylindrical, extreme tip bent dorsally and widened, situated at level of tip of interbase (Fig. 17E). Aedeagal sheath covering basal 2/3 of aedeagus, not distinctly broadened (Fig. 17E). Sperm pump roundish in dorsal view, anterior end situated at level of middle of paramere (Fig. 17D). Ejaculatory apodeme weakly developed (Fig. 17E).

**Female.** Body length 5.0–5.2 mm, wing length 5.7–6.0 mm. Generally resembling male.

***Female terminalia*** (Fig. 18): dark brown, cercus amber-color. Tergites 8 and 9 fused. Cercus relatively slender, upcurved distally, 1.3× longer than tergite 10; hypogynial valve 1.8× as long as sternite 8, gradually narrowed toward tip, basal part 1.3× as wide as that of cercus, tip ending at level of basal 2/3 of cercus (Fig. 18A). Genital frame with genital fork gradually widened toward anterior and posterior ends, narrowest at anterior 1/4, lateral margin of posterior end produced into small lobe; lateral arm of genital fork pointed at tip and curved posteriorly, situated at posterior end of genital fork; sternite 9 relatively long, subacute at tip; one large membranous area present posterior to genital opening (Fig. 18B). Three spermathecal ducts present (Fig. 18B). Spermathecae indistinct.

##### Distribution.

Japan (Honshu) (Fig. 5D) and Russia (FE).

##### Remarks.

This species is similar to a Nearctic species, Ormosia (Parormosia) divergens (Coquillett, 1905) (Coquillett 1905), but is differentiated from it by the following characters: clasper of gonostylus without lobe at fork into dorsal and ventral arms (Fig. 17C) (with roundish lobe at fork in Ormosia (Parormosia) divergens); lobe of gonostylus with short pointed projection at dorsal-apical edge, width of lobe of gonostylus at this point ~ 1/3 length of lobe of gonostylus (dorsal-apical pointed projection long, width of lobe of gonostylus at this point ca. as long as lobe of gonostylus in in Ormosia (Parormosia) divergens).

#### ﻿ Ormosia (Parormosia) phalara

Taxon classificationAnimaliaDipteraLimoniidae

Kato & Kolcsár sp. nov.

CB9BD808-1351-5D18-97E6-B3A267CEE553

https://zoobank.org/65BBAF0A-C395-4E8F-8CF5-D674396F44CA

**Figure 19.**
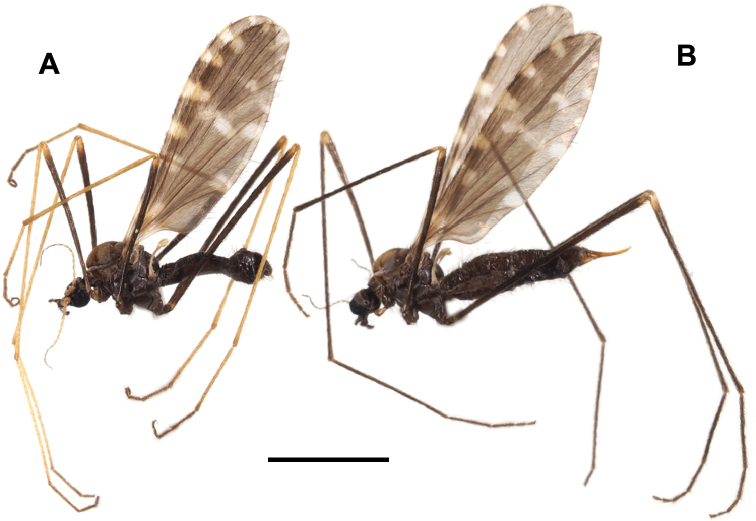
Habitus of Ormosia (Parormosia) phalara Kato & Kolcsár, sp. nov. **A** male **B** female. Scale bar: 2 mm.

**Figure 20.**
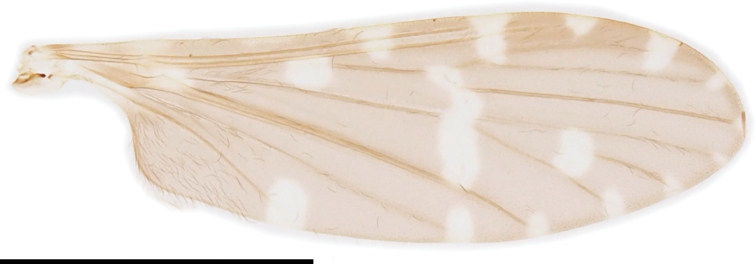
Wing of Ormosia (Parormosia) phalara Kato & Kolcsár, sp. nov. Scale bar: 2 mm.

**Figure 21.**
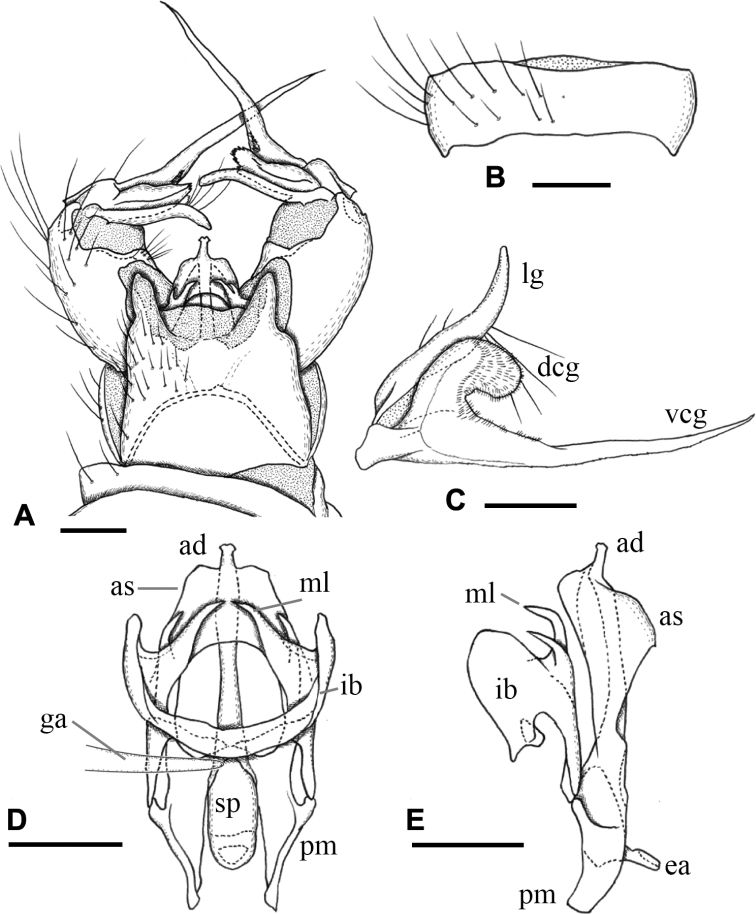
Male terminalia of Ormosia (Parormosia) phalara Kato & Kolcsár, sp. nov. **A** dorsal view **B** sternite 9, ventral view **C** gonostylus, outer surface **D** aedeagal complex, dorsal view (left gonocoxal apodeme omitted) **E** aedeagal complex, lateral view (left = dorsal). Scale bars: 0.1 mm.

**Figure 22.**
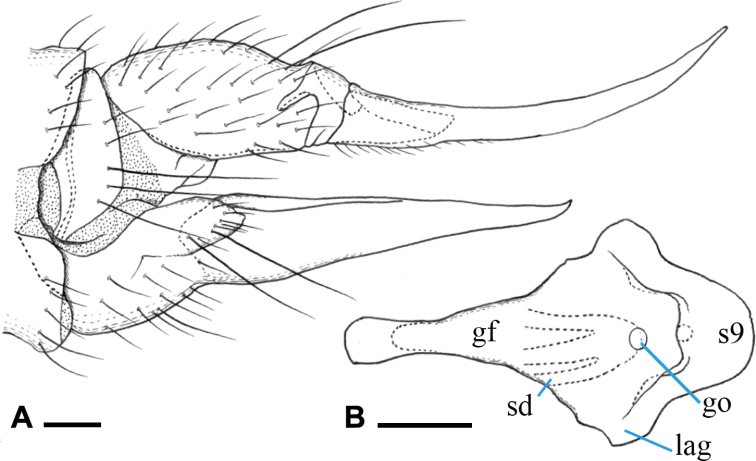
Female terminalia of Ormosia (Parormosia) phalara Kato & Kolcsár, sp. nov. **A** lateral view **B** genital frame, ventral view (left = anterior). Scale bars: 0.1 mm

Figs 5D , 19 , 20 , 21 , 22

##### Type material examined.

***Holotype*.** ♂, pinned. Original label: “JAPAN, Fukuoka, Fukuoka-shi, Sawara-ku, Itaya, Mt. Sefuri-san; alt. 970 m; 10 Jun. 2015, D. Kato leg.” “HOLOTYPE Ormosia (Parormosia) phalara Kato & Kolcsár, sp. nov. [red label]”; BLKU.

***Paratype*s.** Japan • 1♂; Honshu, Aomori, Hirosaki-shi, Ichinowatari-Yamashita; 40.53064°N, 140.44664°E; alt. 173 m; 25 Jul. 2014; light trap; D. Kato leg.; BLKU. • 1 ♀; Honshu, Aomori, Nishimeya-mura, Kawaratai, Ôkawa-rindô Path; 40.50062°N, 140.20405°E; alt. 300 m; 25 Jul. – 6 Aug. 2013; Malaise trap; D. Kato and T. Nakamura leg.; BLKU. • 1 ♂; Honshu, Nagano, Iida, Kamimurahodono; 35.45805°N, 138.01166°E; alt. 1415 m; 3 Aug. 2019; K. Kuroda et al. leg.; EUMJ. • 2 ♂, 1 ♀; Honshu, Nagano; Matsumoto, Azusa lake; 36.12889°N, 137.72512°E; alt. 1000 m; 21 Jul. 2020; L.-P. Kolcsár leg.; CKLP. • 1 ♀ (BOLD ID: JPCOI002-22); Honshu, Nagano, Kiso, Ohara Shinkai; 35.83739°N, 137.77346°E; alt. 1220 m, 19 Jul. 2020; L.-P. Kolcsár leg.; CKLP. • 1 ♂; Honshu, Kanagawa, Hakone; approximate coordinates: 35.23°N, 139.02°E; 25 Jul. 1957; light; S. Hisamatsu leg.; EUMJ. • 3 ♂, 1 ♀; Kyushu, Fukuoka, Fukuoka-shi, Sawara-ku, Itaya, Mt. Sefuri-san; alt. 970 m; 10 Jun. 2015; D. Kato leg.; BLKU.

##### Diagnosis.

General coloration dark brown (Fig. 19). Vertex greyish. Antenna yellowish on pedicel and at least basal segments of flagellum. Mesonotum with ochreous to greyish brown parts. Wing dark brownish tinged, patterned with subhyaline spots on veins, spots free from veins absent. Cell dm closed. Halter yellow. Legs dark brown with narrow yellow areas at tips of femora and bases of tibiae in female, tibiae to tarsi mostly yellow in male. Male terminalia: tergite 9 bearing pair of long membranous lobes at caudal margin. Clasper of gonostylus divided into two arms, ventral arm ~ 3× as long as curved stout dorsal arm, slender blade-shaped, gradually narrow toward tip. Lobe of gonostylus slender and 3/5 length of clasper, tapered distally, with long setae on ventral margin at distal 2/5. Interbase with mesal-apical lobe bearing two claws. Female terminalia with cercus slender, weakly upcurved distally. Lateral arm of genital fork rounded. Sternite 9 rounded posteriorly.

##### Description.

**Male.** Body length 3.2–3.6 mm, wing length 4.2–4.7 mm.

***Head***: covered with yellow and black setae. Vertex grey to dark grey, dark brownish on posterolateral part. Eyes relatively large and widely separated, ~ 4/5 as wide as narrowest point of vertex, ~ 1/2 length of head including rostrum in dorsal view. Rostrum dark brown, ~ 1/2 length of eye in lateral view. Palpus dark brown, 5-segmented, ca. as long as head, palpomere 1 globular and small, palpomeres 2–5 cylindrical, slenderer in palpomeres 2 and 5. Labellum dark brown. Antenna 3× as long as head; 16-segmented, scape dark brown, 2× as long as wide, narrower basally; pedicel pale to dusky yellow, roughly globular, 1/2 of length of scape; flagellomeres pale to dusky yellow, sometimes weakly dark on distal segments, oval on basal one or two segments, long cylindrical on distal segments, each flagellomere with one or two verticils, longest one at most 2× as long as corresponding segment, gradually shorter toward distal segment, sensilla abundant especially in ventral side, at most 1/2 as long as each flagellomere.

***Thorax***: covered with yellow to dark brown setae. Antepronotum dark brown, pale at caudal margin; postpronotum dusky yellow. Mesonotum ochreous to greyish brown, dark brown at anterior and lateral margins, sometimes with three indistinctly dark stripes just anterior to transverse suture. Prescutal pit dark brown, roughly oval with narrower inner end. Tuberculate pit distinct, situated at anterior 1/3 to 1/2 between anterior margin of mesonotum and prescutal pit. Pleuron dark brown, variegated with grey pruinosity (Fig. 19A). Wing (Fig. 20) tinged with dark brown, subhyaline on prearcular region, patterned with subhyaline spots restricted to vicinity of veins, without ones free from veins; spot each at MA, Rs origin, crossvein sc-r, outer end of cell dm, and tips of all longitudinal veins; spot at each tip of R_1_, and R_4_ to CuA smaller; cord seamed with subhyaline; relatively narrow, 3.3× as long as wide; Sc ending at level of R_2_; crossvein sc-r distinct, situated at level of middle of Rs; R_2+3+4_ 2/7 length of R_3_; R_2_ situated between 1/2–1× lengths of itself distal to fork of R_2+3+4_; M_4_ 0.6–0.7× as long as M_3+4_; cell dm closed, 0.7–0.8× as long as cell m_1+2_; wing margin between tips of CuP and A_1_ 2.5–3× as long as that between tips of CuP and CuA; A_1_ curved posteriorly near middle. Halter white to dusky yellow, slightly brownish at base, ~ 1/2 length of thorax (Fig. 19). Legs with coxae dark brown; trochanters dusky yellow on fore pair, brown to dark brown on mid and hind pairs; femora dark brown, bases and tips narrowly yellow; tibiae yellow, tips weakly brownish; tarsi yellow, turning to brown to dark brown toward tip distal to middle of tarsomere 1 (Fig. 19A).

***Abdomen***: dark brown, densely covered with brown setae; yellowish setae present on genital part.

***Male terminalia*** (Fig. 21): Tergite 9 with pair of largely membranous, tongue-shaped lobes at caudal margin, ~ 1/3 length of remainder of tergite 9; tergite 9 slightly longer than wide including caudal lobe (Fig. 21A). Sternite 9 slightly and widely concave at middle of posterior margin (Fig. 21B). Gonocoxite oval, ca. as long as tergite 9, posteroventral margin not produced beyond base of clasper of gonostylus (Fig. 21A). Gonocoxal apodeme long, connected to each other, forming bridge, central part jointed with anteromedial part of interbase (Fig. 21D). Clasper of gonostylus dark, ~ 1.4× longer than gonocoxite, divided into two arms; dorsal arm stout, rounded at tip, curved ventrally in apical view (Fig. 21C) and tip directed distally in dorsal view (Fig. 21A), distal part densely covered with black microscopic setae; ventral arm ~ 3× as long as dorsal arm, slender blade-shaped, gradually narrow toward tip, acute at tip (Fig. 21C). Lobe of gonostylus slender, tapered distally and curved dorsally, 3/5 length of clasper, ventral margin with several long setae at distal 2/5 (Fig. 21C). Interbases fused basally, dorsolateral part roundly produced in lateral view; mesal-apical lobe slender claw-shaped, curved and directed posterodorsally, bearing smaller curved spine arising from ventral surface of mesal-apical lobe (Fig. 21D, E). Paramere wide, distinctly shorter than interbase (Fig. 21D, E). Aedeagus slender and cylindrical, extreme tip and subapical region slightly widened, tip extending beyond tip of interbase (Fig. 21D, E). Aedeagal sheath covering aedeagus except apical part, posterior end ~ 5× as wide as aedeagus (Fig. 21D) at this point and produced dorsally near tip and produced ventrally at distal 3/5 (Fig. 21E). Sperm pump bacilliform in dorsal view, anterior end situated at level of 1/3 of paramere (Fig. 21D). Ejaculatory apodeme developed, dorsoventrally compressed, fin-like plate, ca. as long as diameter of sperm pump (Fig. 21E).

**Female**. Body length 3.6–4.8 mm, wing length 4.2–5.0 mm. Generally resembling male (Fig. 19B) except, antenna shorter, ~ 2× length of head; each flagellomere with ca. eight verticils on each of basal segments, fewer on each of distal segments, longest one at most 1.5× as long as corresponding segment. Tibiae to tarsi dark brown, bases of tibiae narrowly yellow (Fig. 19B).

***Female terminalia*** (Fig. 22): dark brown, cercus and hypogynial valve amber-colored, weakly dark on basal parts. Tergites 8 and 9 fused. Cercus weakly upcurved distally, 1.6× longer than tergite 10; hypogynial valve relatively stout, 1.5× as long as sternite 8, gradually narrowed toward tip, basal part 1.4× as wide as that of cercus, tip ending at level of basal 3/5 of cercus (Fig. 22A). Genital frame with genital fork widened posteriorly, anterior part slender; lateral arm of genital fork weakly produced, rounded, situated at posterior of genital fork; sternite 9 rounded posteriorly; long and arched groove present posterior to genital opening, lateral part curved anteriorly (Fig. 22B). Three spermathecal ducts present, basal parts blackened (Fig. 22B). Spermathecae indistinct.

##### Etymology.

The name of this species, *phalara*, is from the Greek *phalaros*, meaning white-spotted. The name is deemed to be a Latinized feminine adjective in nominative singular.

##### Distribution.

Japan (Honshu and Kyushu) (Fig. 5D).

##### Remarks.

This species resembles a Chinese species, Ormosia (Parormosia) angustaurata Alexander, 1936 (Alexander 1936b), but is distinguished from it by the following characters: scape dark brown (yellow in Ormosia (Parormosia) angustaurata); wing with subhyaline spots restricted to vicinity of veins, without smaller spots free from veins (with at least a few smaller spots free from veins in addition to ones on veins in Ormosia (Parormosia) angustaurata); mesal-apical lobe slender with additional spine near tip (Fig. 21D, E) (stout and without additional spine in Ormosia (Parormosia) angustaurata).

### ﻿Key to Japanese species of Ormosia (Oreophila) and Ormosia (Parormosia)

**Table:** 

1	Wing with cell dm closed or open by atrophy of basal part of vein M_3_; if cell dm closed, male terminalia with clasper of gonostylus simple, not forked near base	**subgenus Ormosia s. s.**
–	Wing with cell dm closed or open by atrophy of crossvein m-m; if cell dm closed, male terminalia with clasper of gonostylus bifid	**2**
2	Cell dm closed (Figs 13, 20) or open (Fig. 16); clasper of gonostylus forked near base, dorsal arm stout and curved, distinctly shorter than ventral arm (Figs 14C, 17C, 21C)	**3 (subgenus Parormosia)**
–	Cell dm open (Figs 2, 9); clasper of gonostylus simple (Figs 3C, 7C), or forked near base with dorsal arm slender and nearly as long as ventral arm (Fig. 10C)	**5 (subgenus Oreophila)**
3	Wing dark, with white maculae/spots (Fig. 20)	**Ormosia (Parormosia) phalara Kato & Kolcsár, sp. nov.**
–	Wing unpatterned except barely with dark stigma (Figs 13, 16)	**4**
4	Male flagellomeres brown to dark brown; mesonotum subnitidous; cell dm open (Fig. 16); tibiae dark brown in both sex (Fig. 8B); interbases fused medially into large roundish plate, without mesal-apical lobe (Fig. 17D)	**Ormosia (Parormosia) nippoalpina Alexander**
–	Male flagellomeres yellowish on basal segments; mesonotum not nitidous; cell dm usually closed (Fig. 13); tibiae yellowish in male (Fig. 12A) and dark brown in female (Fig. 12B); interbase with mesal-apical lobe (Fig. 14D)	**Ormosia (Parormosia) diversipes Alexander**
5	Body almost entirely brownish black (Fig. 8A); clasper of gonostylus divided (Fig. 10C); cercus stout, strongly upcurved (Fig. 11A)	**Ormosia (Oreophila) sootryeni Lackschewitz**
–	Body entirely yellowish or pale brownish (Figs 1, 6); clasper of gonostylus undivided (Figs 3C, 7C)	**6**
6	Male antenna ca. as long as or slightly longer than body (Figs 1B, 6); subapterous in male (Figs 1B, 6); distal part of clasper of gonostylus ca. as wide as lobe of gonostylus (Fig. 7C); female unknown	**Ormosia (Oreophila) komazawai Kato & Kolcsár, sp. nov.**
–	Antenna distinctly shorter than 1/2 length of body; wing fully developed (Fig. 1A); distal part of clasper of gonostylus 2× as wide as lobe of gonostylus (Fig. 3C)	**Ormosia (Oreophila) confluenta Alexander**

## Supplementary Material

XML Treatment for Ormosia

XML Treatment for Subgenus Oreophila

XML Treatment for Ormosia (Oreophila) confluenta

XML Treatment for Ormosia (Oreophila) komazawai

XML Treatment for Ormosia (Oreophila) sootryeni

XML Treatment for Subgenus Parormosia

XML Treatment for Ormosia (Parormosia) diversipes

XML Treatment for Ormosia (Parormosia) nippoalpina

XML Treatment for Ormosia (Parormosia) phalara
